# Ceftiofur-resistant *Salmonella enterica* serovar Heidelberg of poultry origin – a risk profile using the Codex framework

**DOI:** 10.1017/S0950268819001778

**Published:** 2019-11-04

**Authors:** Carolee Carson, Xian-Zhi Li, Agnes Agunos, Daleen Loest, Brennan Chapman, Rita Finley, Manisha Mehrotra, Lauren M. Sherk, Réjean Gaumond, Rebecca Irwin

**Affiliations:** 1 Centre for Food-borne, Environmental and Zoonotic Infectious Diseases, Public Health Agency of Canada, Guelph, Ontario, Canada; 2 Veterinary Drugs Directorate, Health Products and Food Branch, Health Canada, Ottawa, Ontario, Canada; 3 National Microbiology Laboratory, Public Health Agency of Canada, Guelph, Ontario, Canada; 4 University of Guelph, Guelph, Ontario, Canada; 5 Market and Industry Services Branch, Agriculture and Agri-Food Canada, Ottawa, Ontario, Canada

**Keywords:** Antimicrobial resistance in agricultural settings, food-borne zoonoses, public health, risk assessment, *Salmonella*

## Abstract

Codex published the ‘Guidelines for Risk Analysis of Foodborne Antimicrobial Resistance’ to standardise the approach for evaluating risk posed by foodborne antimicrobial-resistant bacteria. One of the first steps in the guidelines is to compile a risk profile, which provides the current state of knowledge regarding a food safety issue, describes risk management options and recommends next steps. In Canada, ceftiofur/ceftriaxone-resistant *Salmonella enterica* subsp. *enterica* serovar Heidelberg from poultry was identified as an antimicrobial resistance (AMR) food safety issue. The first objective of this article was to contextualise this food safety issue, using the risk profile format of the Codex Guidelines. A second objective was to evaluate the applicability of the Codex Guidelines. This risk profile indicated that ceftiofur/ceftriaxone-resistant *S*. Heidelberg (CSH) was commonly isolated from poultry and was associated with severe disease in humans. Ceftiofur use in poultry hatcheries temporally mirrored the prevalence of CSH from poultry meat at retail and from people with salmonellosis. The evidence was sufficient to indicate the need for risk management options, such as restricting the use of ceftiofur in poultry. The Codex Guidelines provided a useful approach to summarise data for decision-makers to evaluate an AMR food safety issue.

## Introduction

Antimicrobial resistance (AMR) is a worldwide health threat, as demonstrated by global AMR surveillance data [1]. Although the most significant human health impacts associated with AMR occur in the human clinical setting [2], impacts from human exposure to resistant bacteria from other sources, such as from animals or food, play an important role [3–5]. In Canada, in 2017, ~77% of the antimicrobials distributed or sold for use in people, animals and crops were intended for use in production animals (including poultry) [6]. However, quantification of the association between AMR arising through the food chain on adverse human health impacts has proven to be challenging and has been the subject of debate for decades. This is due in part to the complexity of multiple foodborne AMR transmission pathways, the exchange of resistance determinants between bacterial species and the lack of standardised methods to conduct an evaluation of the risk.

To partly address the lack of a standardised approach, the Codex Alimentarius Commission adopted the ‘Guidelines for Risk Analysis of Foodborne Antimicrobial Resistance’ [7] (herein denoted the ‘Codex Guidelines’). One of the first steps in these guidelines is to complete a risk profile, which is intended to provide the current state of knowledge regarding a food safety issue and describe possible risk management options [7]. Based on the findings of a risk profile, subsequent steps in risk analysis are either to make an immediate (and/or provisional) decision for risk management, launch a full qualitative or quantitative risk assessment, maintain *status quo* or gather more data before making a preliminary decision [7].

Throughout this article, the definitions for the AMR food safety issue and AMR hazard of concern from the Codex Guidelines have been applied [7]; note that these definitions can be different from the common or colloquial usage of these terms. ‘Issues’ and ‘hazards’ in Codex do not equate to ‘risk’, unless determined to be so according to the findings of the risk profile or risk assessment.

For this risk profile, the AMR food safety issue under evaluation was ceftiofur-resistant *Salmonella enterica* subsp. *enterica* serovar Heidelberg (*S*. Heidelberg) arising from poultry, as identified by the Canadian Integrated Program for Antimicrobial Resistance Surveillance (CIPARS). Our attention was on this particular AMR food safety issue for several reasons. First, ceftiofur is a 3^rd^ generation cephalosporin (3GC), a class of antimicrobials considered critically important to human medicine [8, 9]. Although ceftiofur is only registered for veterinary use, the 3GCs used in human medicine are related, such as ceftriaxone. Resistance to one advanced cephalosporin is closely associated with resistance to another and resistance to ceftiofur confers (almost 100%) resistance to ceftriaxone [10]. Second, the bacterial strain under evaluation was *S.* Heidelberg, because it is a serovar that tends to cause more severe illness than other *Salmonella* serovars [11]. *S.* Heidelberg affects vulnerable segments of the human population, including pregnant women and children, for whom the 3GCs are one of the limited antimicrobials available for treatment [12]. Finally, the food commodity under evaluation was poultry as it is a meat source commonly consumed by Canadians and is a source of *S.* Heidelberg [13]. To date, there have been interventions to address this AMR food safety issue, but describing the issue using formal risk assessment methodology and evaluating whether additional measures should be taken from a risk perspective, had yet to be conducted.

The major objective of this article was to describe and contextualise the AMR food safety issue of ceftiofur/ceftriaxone-resistant *S.* Heidelberg (CSH) from consumption of poultry in Canada, using the risk profile format of the Codex Guidelines [7]. The second objective was to use this AMR food safety issue to critically evaluate the Codex Guidelines in terms of their applicability for constructing a risk profile.

## Materials and methods

Data sources included published surveillance data from CIPARS, peer-reviewed literature, grey literature, expert opinion and demographic information as published by Statistics Canada or Agriculture and Agri-Food Canada. The data were collated and reported according to the headings in the Codex Guidelines' Appendix 1: Elements for Consideration in a Foodborne Antimicrobial Resistance Risk Profile [7]. Unless stated otherwise, all information pertains to Canadian data and the Canadian context. Information from international sources was included when Canadian data were lacking.

Where appropriate, and for summarising the results for uptake by policy makers, the data for each major section of the risk profile were categorised into levels of concern in reference to other antimicrobials, other foodborne antimicrobial-resistant pathogens and other food animal species, with scores of 1, 2 and 3 (1 = lowest concern; 3 = highest concern). There are no current standards, national, international, or otherwise, for these levels of concern; however, these qualitative indicators are defined as transparently as possible for each section in this article. Additionally, a measurement of data quality/relevance was provided for each section of the risk profile using the following criteria and scoring: (1) applicability of the data within a Canadian context based on the location of information collection (Canada = 3, United States = 2, other country = 1 and data gap = 0); (2) type of information (active surveillance = 5, passive surveillance = 4, large scale peer reviewed publication = 3, small scale peer reviewed publication = 2, empirical information = 1 and data gap = 0) and (3) year of data collection (⩾2015 = 4, 2010–2014 = 3, 2006–2009 = 2, <2006 = 1 and data gap = 0). Scores were summed to provide an overall measure of data quality. Higher summed scores indicated higher quality data and better applicability to the current evaluation of risk. The scoring for the level of concern and data quality/relevance was completed by two of our research team members.

Where appropriate and informative or when *S.* Heidelberg specific data were lacking, data for other *Salmonella* serovars and generic *Escherichia coli* were included. *Salmonella* spp. and *E. coli* are close relatives in the same family of *Enterobacteriaceae,* often sharing similar mechanisms of resistance. Shared plasmids may include those that impart resistance to *β*-lactams including cephalosporins [10, 14]. Generic *E. coli* is considered a valuable indicator for measuring the pool of resistance elements available for exchange with other bacterial species.

## Results (headings as per the Codex Guidelines)

### Description of the AMR food safety issue

According to the Codex Guidelines, an AMR food safety issue is the defined combination of three components: the resistance hazard of concern (i.e. the resistant microorganism(s) and/or determinant(s) of resistance), the antimicrobial agent to which resistance is expressed and the food commodity in which the hazard is identified [7]. For this risk profile, the AMR food safety issue was 3GC-resistant *S.* Heidelberg arising from poultry. In addition to the rationale provided in the Introduction, further reasons for selecting this combination are as follows. The rationale for selecting resistance to 3GCs, is that this is considered as one of the ‘fastest emerging resistance problems worldwide’ [15]. Part of the rationale for selecting *Salmonella* was that the most recent data from the Canadian National Enteric Disease Surveillance Program indicated that in 2016, *Salmonella* was the most common enteric pathogen submitted to this program, with an incidence rate of 21 laboratory-confirmed cases per 100 000 people [16]. Between 2000 and 2010, ~88 000 cases of domestically acquired, foodborne non-typhoidal *Salmonella* (NTS) occurred annually in Canada, leading to ~925 hospitalisations (8% of all hospitalisations due to foodborne illness) and 17 deaths (7% of all deaths due to foodborne illness) [17, 18]. In comparison to all drug-resistant bacterial infections, drug-resistant NTS are one of the priority organisms identified for surveillance by the World Health Organisation [19] and are considered a ‘Serious Threat’ by the US Centres for Disease Prevention and Control (CDC) [2]. A Canadian AMR threat assessment ranked drug-resistant *Salmonella* as a Tier 3 pathogen (of four Tiers with Tier 1 being the most critically important) [20]. For the selection of the particular serovar, from 2014–2016 *S*. Heidelberg was the third most common *Salmonella* serovar isolated from Canadians who were sick and had a sample submitted for laboratory testing [16]. *S.* Heidelberg is a relatively more prevalent serovar in North America and Africa compared to other areas of the world [16, 21, 22, 23] (Supplementary Table S1). It is also not a new serovar and has been identified as early as 1962 in poultry and poultry products in Brazil [24].

**Fig. 1.**
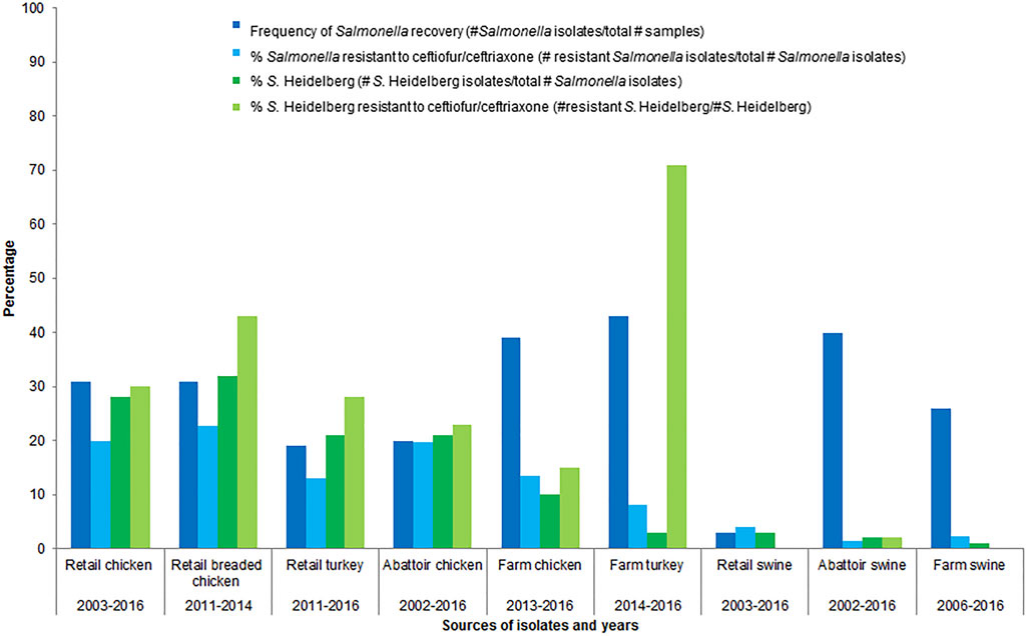
Percentages of isolates recovered and percentages of *Salmonella* spp. and *S*. Heidelberg isolates resistant to ceftiofur/ceftriaxone, by food animal species.

Further rationale for choosing poultry as the food animal commodity of concern is that poultry is consumed in Canada more so than beef or pork [25, 26]; of the food animal commodities sampled by CIPARS, poultry was the predominant sector from which *S.* Heidelberg was isolated (Fig. 1) [13, 27, 28]. The prevalence of ceftiofur resistance was higher in *Salmonella* and in *S*. Heidelberg isolates from healthy poultry/poultry meat than from the other healthy food animals/meat sampled by CIPARS (Fig. 1) [13, 27–39]. Ceftiofur resistance in *Salmonella* has been detected in clinical isolates from sick cattle and sick horses; sick animals are unlikely to enter the food chain. *Salmonella* is rarely detected in retail pork and beef in Canada.

#### Summary level of concern and data quality

To summarise the information of this section, the antimicrobial is in a class of critical importance to human medicine, *S.* Heidelberg is of concern to human health, and frequently consumed poultry is the food animal sector where the 3GC resistance and the serovar were predominantly found. The level of concern is considered three and the data quality score is 12 (Canadian data = 3, active surveillance = 5, ⩾2015 = 4).

### Information on the antimicrobial resistant organism and/or AMR determinant

#### Characteristics of the identified foodborne microorganism

##### Sources and transmission routes

This risk profile focussed on foodborne transmission; 80% of NTS cases in Canada are estimated to be foodborne [17, 40]. Non-foodborne transmission of *S*. Heidelberg, though rare, has been documented, such as transmission by direct human-to-human contact, nosocomial transmission and possibly via direct contact between animals and humans [41, 42]. *S.* Heidelberg has also been transmitted via cross-contamination of food products in the kitchen (e.g. contamination of tomato salad prepared on the same table as raw chicken) [43].

In addition to the CIPARS data (Fig. 1) indicating poultry being the primary reservoir of *S*. Heidelberg of the sampled food/animals, in a Canadian case-control study, 34% of all human *S*. Heidelberg infections were attributable to eating chicken strips or nuggets and 16% were attributable to consuming eggs [44]. Within poultry, *S.* Heidelberg has been isolated from meat and faeces [27], crops of birds, reproductive tracts of hens (ovaries and oviducts), eggs and eggshells [45–50].

##### Pathogenicity, virulence and linkage to resistance of particular strains

Pathogenicity refers to the ability of an organism to cause disease and virulence to the severity of that disease [51]. The available data did not clearly differentiate between pathogenicity and virulence; as such, the Codex Guidelines subheadings for pathogenicity and virulence were combined. Although not requested in the Codex Guidelines, information regarding pathogenicity and virulence of CSH both in the animal species of interest (poultry) and in humans, was provided.

In poultry, *S.* Heidelberg displays variable pathogenicity and virulence. It can cause diseases such as peritonitis, hepatitis, pericarditis, pneumonia and enteritis, with mortality rates of up to 13% [52]. A study of broiler chicks in Brazil demonstrated damage to intestinal mucosa similar to that caused by *Salmonella enterica* subsp. *enterica* serovar Enteritidis, indicating that *S*. Heidelberg could have importance as a pathogen in young chicks [24].

While asymptomatic human cases have occurred [53, 54], *S.* Heidelberg is generally considered pathogenic to humans. *S.* Heidelberg has been described as being ‘disproportionately associated with invasive infections and mortality in humans’ [55]. Infections may result in mild to moderate illness, but *S.* Heidelberg can also cause severe illness, with complications such as septicaemia and myocarditis [56]. In Canada, in 2016, a larger proportion of *S.* Heidelberg from 2405 NTS isolates was obtained than the other NTS serovars in human blood and urine (sources that signify invasive infections), with 17% and 6% of 315 *S.* Heidelberg isolates, respectively, from blood and urine [13].

At the molecular level, virulence genes, located on chromosomes and plasmids, including those contained in *Salmonella* pathogenicity islands (on chromosomes), have been identified in *Salmonella*, such as *S.* Heidelberg [55–57]. These genes encode factors to facilitate endothelial uptake, as well as regulatory and effector virulence factors for adhesion, invasion and toxin production [58]. For example, in addition to the presence of multidrug resistance plasmids, *S.* Heidelberg isolates, including those from ground turkey-associated outbreak in the USA, were found to carry a variety of phages (such as prophages P22, Gifsy-2, P4 and P2-like), virulence genes including 62 pathogenicity and 13 fimbrial markers and/or IncX plasmids [56]. The virulence factors can contribute to *Salmonella* colonisation and persistence [56, 59]. Moreover, the co-presence of AMR and virulence genes has been demonstrated in *S*. Heidelberg isolated from broiler chickens [58]. The IncA/C plasmids, found in *S.* Heidelberg, have been found to encode up to eight resistance genes, including those imparting ceftriaxone resistance [56]. The simultaneous presence of multiple plasmids, including IncA/C, IncFIB and IncX (VirD4/B4), that contain resistance genes and/or virulence genes have been observed in *S*. Heidelberg of porcine and turkey origin in the USA [55, 56].

##### Distribution, frequency and concentrations of the AMR hazard(s) in the food chain

In Canada, *S.* Heidelberg has consistently been one of the top three serovars isolated from chicken at retail, at slaughter and from clinical isolates from chickens [13, 39]. Although the serovar types isolated from retail turkey vary considerably from year to year, *S.* Heidelberg and *S.* Enteritidis were consistently the top two serovars [13, 27, 28, 38, 39].

Based on CIPARS data, there has been a decline in the overall recovery of *Salmonella* from retail chicken. The average percentage of isolates recovered compared to the samples taken (percent recovery), across participating provinces, peaked in 2009 at 43% and decreased to 28% in 2016 [13]. The percent recovery of *Salmonella* and *S.* Heidelberg at different sampling points in the food chain also varies. For example, the percent recovery of *Salmonella* from chicken(s) (number of *Salmonella* isolates/total number of samples) for 2016 varied from 46% at the farm, to 14% at slaughter and to 28% at retail [13]. The corresponding percent recovery of *S.* Heidelberg (number of *S*. Heidelberg isolates/total number of *Salmonella* isolates) was 1% at farm, 5% at slaughter and 3% at retail [13]. It is unknown why there was a decrease in the recovery rate for *Salmonella* from the farm to slaughter and then an increase from slaughter to retail, or why this trend is the opposite for *S.* Heidelberg. For 2016, the percentage of *S.* Heidelberg resistant to ceftiofur/ceftriaxone was 4% at farm, 18% at slaughter and 12% at retail [13]. These data suggest a potential amplification of *S*. Heidelberg, and a potential amplification of resistance in *S*. Heidelberg, between farm and retail. Statistical significance testing of these point prevalence differences would be useful.

While Canadian data on the frequency of occurrence of *S*. Heidelberg and frequency of resistance to ceftiofur are available, the concentration of *S.* Heidelberg (number of colony forming units/gram of sampled material) at various stages throughout the food chain represents a large data gap; this information would be required for quantitative modelling of the hazard.

**Fig. 2.**
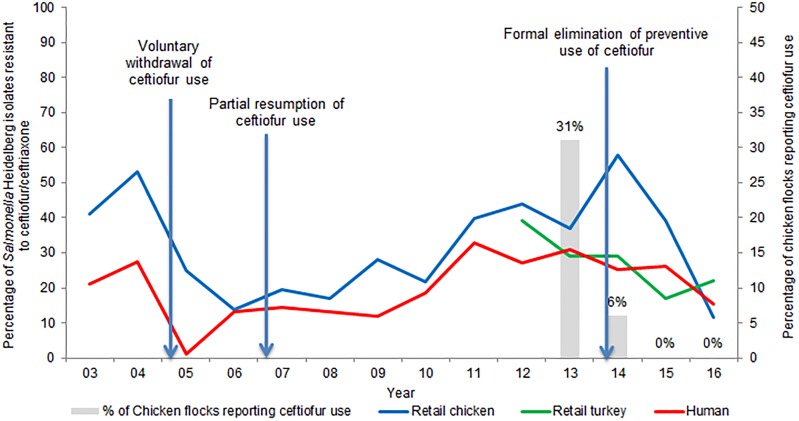
Percentage of ceftiofur/ceftriaxone-resistant *Salmonella* Heidelberg from retail poultry and humans, and ceftiofur use in chicken flocks.

Supplementary Table S2 presents the temporal trends in the frequency of ceftiofur resistance among the most common serovars isolated from sampled retail poultry and people in Canada. The frequency of ceftiofur/ceftriaxone resistance among *S.* Heidelberg isolates from retail chicken over time has varied, from 41% in 2003, to 14% in 2006 and peaking at 58% in 2014. On average, from 2012 to 2015, 29% of *S.* Heidelberg isolated from retail turkey meat was resistant to ceftiofur. Ceftiofur/ceftriaxone resistance among *S.* Heidelberg isolates from retail poultry and from human clinical cases follow similar trends, as is illustrated in Figure 2. The average number of *S.* Heidelberg isolates identified was 71 from retail chicken (2003–2016), 29 from retail turkey (2011–2016) and 417 from human clinical cases (2003–2016); hence, more statistical power exists to detect temporal changes in ceftiofur/ceftriaxone resistance among CSH isolates of human origin. A decrease in 2005 of ceftiofur resistance and a subsequent increase reflects changes in ceftiofur use practices in Canadian hatcheries (more details are included in ‘Effectiveness of current management practices in place based on surveillance data or other sources of information’).

As shown in Supplementary Table S2, *Salmonella enterica* subsp. *enterica* serovar Kentucky from poultry is frequently resistant to ceftiofur. However, *S*. Kentucky is rarely isolated from Canadians; from 2011 to 2015, 62 isolates were submitted for susceptibility testing, with many thought to be travel related. Of the human *S*. Kentucky isolates, only five were resistant to *β*-lactams. A Canadian study has reported high-sequence identity (95–99%) of *bla*_*CMY−2*_ containing IncI1 plasmids from *S*. Heidelberg isolates of Canadian human and poultry sources in comparison with the IncI1 plasmid of an *S*. Kentucky from chicken product in the USA, suggesting a common reservoir and intra-species dissemination for ceftiofur/ceftriaxone resistance determinants in *Salmonella* spp. [58].

The regional frequency of CSH varies from year to year. In 2013, the frequency of ceftiofur/ceftriaxone resistance among *S.* Heidelberg from retail chicken ranged from 26% to 80% across the provinces participating in CIPARS (British Columbia > Québec > Maritime Provinces (New Brunswick, Nova Scotia and Prince Edward Island) > Saskatchewan > Ontario) [39]. In 2016, the percentage of isolates resistant to ceftiofur ranged from 0% (British Columbia and Ontario) to 15% in Québec. The reason for this regional variation is unclear, however from an analysis perspective, there are often very small numbers of *S.* Heidelberg isolated each year in some provinces [13, 39].

##### Growth and survivability, including inactivation in foods (D-value, minimum pH for growth) of the foodborne antimicrobial-resistant microorganism(s) in the food commodity production-to-consumption continuum

Inactivation factors such as the *D*-value (i.e. the time for a one log reduction in *Salmonella* numbers from an initial set concentration in log/mL) and minimum pH for growth are necessary to describe the growth and survivability of pathogens throughout the production-to-consumption continuum. Therefore, two original Codex subheadings (i.e. Growth and survivability of foodborne antimicrobial resistant microorganism(s) in the food commodity production to consumption continuum and Inactivation in foods (e.g. *D*-value and minimum pH for growth)) were combined.

Selection for resistant serovars takes place during the pre-harvest stage, due to antimicrobial use (AMU) and other farming practices. However, there are various points along the production-to-consumption continuum, both pre- and post-harvest, where practices can have an impact on the colonisation, prevalence and growth of *Salmonella* (i.e. pathogen load). Specific risk factors are discussed in ‘Description of the food production to consumption continuum (e.g. primary production, processing, storage, handling, distribution and consumption) and the risk factors that affect the microbiological safety of the food product of concern’ and risk mitigation options are elaborated upon in ‘Risk Management Information’. Thus far, studies have been unable to demonstrate that AMR, including ceftiofur/ceftriaxone resistance, changes *Salmonella*'s ability to survive various slaughter and food preparation practices. For example, AMR did not have an effect on *Salmonella*'*s* ability to withstand chlorination in chilled water during processing at an abattoir, or its ability to withstand heat during food preparation [60]. At a concentration of 30 ppm residual chlorine (with a pH of 6 and at 4 °C), resistance had no effect on the survival of *Salmonella enterica* subsp. *enterica* serovar Typhimurium (assuming that *S.* Heidelberg would behave similarly) in an artificial medium, as measured by changes in *D*-values [61]. The *D*-values ranged from 3.8–4.3 min, and did not differ between control and test groups [61]. This is consistent with *D*-values reported for *S.* Typhimurium *in situ*, of 3.4 and 5.8 min (30 ppm residual chlorine in chilled water) following 0 and 8 h of processing respectively [62].

#### Characteristics of the resistance expressed by the antimicrobial resistant microorganism(s) and/or AMR determinant(s)

##### Resistance mechanisms and location of the AMR determinants

Cephalosporin resistance is attributable to multiple mechanisms including inaccessibility of the drug to its targets (due to reduced membrane permeability and drug efflux), target alterations in penicillin-binding proteins and drug inactivation by *β*-lactamases. To date, *β*-lactamases have been considered the most frequent and important mechanism of cephalosporin resistance in Gram-negative bacteria [10, 63]. However, recent work has demonstrated that repurposed metabolic enzymes can decrease the susceptibility of *S.* Enteritidis to ceftiofur, independent of genetically driven *β*-lactamase activity [64].

When resistance to 3GC is due to *β*-lactamases, it is due to class C AmpC *β*-lactamases (e.g. CMY-2 – a cephalomycinase encoded by the *bla*_CMY_ gene), class A extended-spectrum *β*-lactamases (ESBLs, such as certain TEM, SHV and CTX-M) or class A, B or D carbapenem-hydrolysing *β*-lactamases (carbapenemases) [10, 63]. Genes encoding these enzymes are generally located on plasmids. However, the integration of plasmidborne *bla*_CMY_ gene into the chromosome of *S*. Heidelberg has been observed [58].

In isolates of animal origin, CMY enzymes are frequent globally, while ESBLs are emerging. In Canada, the analysis of animal and human NTS isolates from 2010 and 2011 found *bla*_CTX−M_ genes in five (0.7%) isolates from turkeys, and in two (0.1%) isolates from humans. Among CMY enzymes, CMY-2 is the most frequently identified variant in *Salmonella* (including *S.* Heidelberg) [65–68]. Genetically similar CMY-2 plasmids in *Salmonella* (of human origin) and *E. coli* (of human, animal and environmental origin) are distributed widely across Canada [69]. Another Canadian study supports these findings, but also identified CMY-2 plasmids primarily isolated from poultry [70].

##### Cross-resistance and/or co-resistance to other antimicrobial agents

Cephalosporin use, specifically ceftiofur, the only 3GC available for food animals, is considered to be a major driver of selection and development of 3GC resistance in food animals [67, 71–74]. Particularly in Canada, the majority of CSH poultry isolates displayed cross-resistance to other *β*-lactams, but little phenotypic co-resistance to antimicrobials in other classes (Supplementary Table S3). Between 2003 and 2016, for isolates from chicken, only nine (3%) CSH were identified as resistant to three or more antimicrobial classes, with eight of these identified prior to 2012. Six CSH (67%) were resistant to three antimicrobial classes: three were resistant to *β*-lactams, aminoglycosides and sulfonamides; two were resistant to *β*-lactams, aminoglycosides and tetracyclines and one was resistant to *β*-lactams, sulfonamides and chloramphenicol. One isolate was resistant to four classes (*β*-lactams, aminoglycosides, tetracyclines and sulfonamides) and one isolate to five classes (*β*-lactams, aminoglycosides, tetracyclines, sulfonamides and chloramphenicol). Turkey retail surveillance identified five CSH (9%) with resistance to three or more classes of antimicrobials, one from 2011, three from 2012 and one from 2013. Four of these CSH were resistant to four antimicrobial classes (*β*-lactams, aminoglycosides, tetracyclines and sulfonamides). The remaining isolate demonstrated the same resistance pattern, except for sulfonamides.

A Canadian study assessed the presence of plasmids among *Salmonella* (including *S.* Heidelberg) and *E. coli* of poultry, bovine and swine origin [70]. Plasmids encoding only resistance to *β*-lactams, including ceftiofur (with no co-resistance to chloramphenicol, gentamicin, kanamycin, trimethoprim-sulfamethoxazole and tetracycline), were found primarily in poultry products, suggesting selection pressure of *β*-lactam use in poultry on bacterial resistance [70]. Genetic analysis of *S*. Heidelberg broiler chicken isolates in Canada has identified a chromosomal gene conferring fosfomycin resistance [75].

##### Transferability of resistance determinants between microorganisms

Transferable resistance determinants mediating *β*-lactamase production and fluoroquinolone resistance have been observed in human and animal *Salmonella* isolates [76, 77], and *Salmonella* genomic islands have been identified as mobile genetic elements containing integrons and clusters of resistance and/or virulence genes [78–80]. Among *S.* Heidelberg, dissemination of AMR determinants is likely due to plasmid-mediated conjugative transfer and by transposons [81]. Both *S.* Heidelberg and *S.* Kentucky possess a propensity to acquire and disseminate multiple plasmids encoding for multidrug resistance [82]. Whole genome sequencing (WGS) of *S.* Heidelberg from both human and poultry sources in Canada demonstrated a close relationship between *bla*_CMY−2_ containing plasmids, suggesting horizontal plasmid dissemination, rather than just clonal spread of a particular *S.* Heidelberg strain [58]. The most frequently-isolated *bla*_*CMY−2*_ carrying plasmids of *S.* Heidelberg isolates include IncI1 (e.g. subtyping ST12 and ST25) and IncA/C (ST2), which have likely contributed to the widespread distribution of CSH and other 3GC-resistant NTS serovars from human and animal sources [58, 83–85].

Transduction-mediated transfer of resistance genes (including the CMY-2 gene) from *S*. Heidelberg to *S*. Typhimurium has been demonstrated [86]. A recent US study found that bacteriophage-mediated AMR gene transfer among *S.* Heidelberg might occur more readily on turkey farms than on chicken farms [81]. Another study's findings suggest that *S.* Heidelberg may acquire fitness enhancing ColE1 plasmids (associated with decreased susceptibility to aminoglycosides and fosfomycin) through poultry litter, although the bacterial plasmid donors have yet to be identified [87].

Conjugative transfer of CMY-2-encoding plasmids from *E. coli* to *Salmonella* was demonstrated using turkey poults (under laboratory conditions) [88], between animal-associated *Salmonella* and *E. coli*, in addition to findings that suggest transmission of a CMY-2 plasmid from food animals to humans [89].

#### Summary level of concern and data quality

This section contains many different data elements, which proves challenging to come up with a summary measure of the level of concern and data quality. The level of concern was estimated at three, as *S.* Heidelberg is associated with higher morbidity in humans than other serovars, is predominantly transmitted through food sources, resistance determinants have been linked with virulence determinants, it possesses genetically transferable resistance (i.e. predominantly plasmid-mediated) and genetically similar isolates have been found in chicken and in people. One moderating factor is that ceftiofur resistance is not frequently expressed with resistance to other classes of antimicrobials in the Canadian isolates tested. The available data are Canadian, recent, and from active surveillance, resulting in an overall data quality for this section of 12.

### Information on the antimicrobial agent(s) to which resistance is expressed

#### Class of the antimicrobial agent(s)

Cephalosporins are *β*-lactam antimicrobials, and are bactericidal via inhibition of bacterial cell wall synthesis. They are divided into five generations based on their spectrum of activity, including *β*-lactamase stability and pharmacological properties; generally, each generation has advantages over the previous generation(s). The 3GCs have increased activity against Gram-negative bacteria, such as *Enterobacteriaceae*, but decreased activity against Gram-positive bacteria [90].

#### Non-human uses of the antimicrobial agent(s)

Ceftiofur was developed strictly for veterinary use and, since the early 1990s, has been the only 3GC authorised for use in specific livestock in Canada. Ceftiofur has never been approved for use in chickens in Canada; any use in chickens is considered extra-label use (ELU). An earlier authorised indication for ceftiofur use in day-old turkey poults was withdrawn in 2012.

##### Formulation of the antimicrobial agent(s)

Three salt formulations of ceftiofur (ceftiofur sodium, ceftiofur hydrochloride and ceftiofur crystalline free acid) are available in Canada for parenteral administration. Depending on animal species or indication, ceftiofur can be administered intra-muscularly, subcutaneously or intra-mammary [91].

##### Distribution, cost and availability of the antimicrobial agent

Ceftiofur is only available by veterinary prescription in Canada and costs are borne by producers.

In broiler poultry, 1 mL of ceftiofur sodium at 50 mg/ml could be enough for 250–625 chicks (0.08–0.20 mg/chick), at an approximate cost of less than one Canadian cent per chick/egg.

##### Purpose and use of the antimicrobial agent(s) in feed, food animals, crop production and/or during food processing AND Potential extra-label/off label, use of approved antimicrobial agent(s) and use of non-approved antimicrobial agent(s)

Two of the Codex subheadings were combined to eliminate duplication of information.

Ceftiofur is licensed in Canada for the treatment of bacterial respiratory disease (bovine, porcine, equine and ovine), bovine acute interdigital necrobacillosis, post-partum metritis, mastitis and treatment of urinary tract infections in dogs [91]. Ceftiofur is not available for use in feed, water, crop production or during food processing.

In Canada, ceftiofur has been used in an extra-label manner in poultry to prevent or control diseases such as omphalitis and yolk sac infections, caused by avian pathogenic *E. coli* (APEC) and other *Enterobacteriaceae* [92]. APEC-associated neonatal diseases are the most frequently diagnosed and economically important diseases of broilers and turkeys in Canada. Ceftiofur administration establishes sufficient circulating metabolites in the blood and yolk sac of chicks prior to their exposure to bacterial pathogens in the barn [93].

##### Methods, routes of administration of the antimicrobial agent(s) (individual/mass medication, local/systemic application) and frequency

Detecting and treating individual sick birds is not cost-effective in a commercial hatchery setting, and mass medication *in-ovo,* or to day old chicks, has been perceived as a practical approach to preventing disease [92]. In poultry, ceftiofur has been administered *in-ovo,* though subcutaneous injections to day-old chicks could also have occurred. Ceftiofur has been combined with hatchery vaccines (i.e. vaccination for Marek's Disease), which is typically administered at approximately day 18 of incubation [94]. Ceftiofur could be administered to elite breeding stocks to prevent or treat APEC, or other susceptible pathogens, which may potentiate vertical transmission of resistant strains [95].

##### Potential role of cross-resistance or co-resistance with use of other antimicrobial agent(s) in food production

Based on CIPARS data from sentinel broiler chicken farms, the only other *β*-lactam antimicrobial used is penicillin [28]. As *Salmonella* spp. and *E. coli* are intrinsically resistant to penicillin, penicillin use is not expected to contribute to ceftiofur cross-resistance.

From 2014–2016 in Canada, CSH isolates of chicken origin were infrequently co-resistant to streptomycin and/or tetracycline. During this period, streptomycin use was not reported and only 1% of broiler flocks sampled reported tetracycline use [13, 27, 28]. Given the infrequency of use, it is unlikely that these drugs are driving factors in the development of resistance to ceftiofur.

##### Trends in the use of the antimicrobial agent(s) in the agricultural and aquaculture sectors and information on emerging resistance in the food supply

The information in this section was limited to ceftiofur use trends in poultry, and related resistance information. Comprehensive information on emerging resistance was better situated in ‘Risk Management Information’.

A study of Québec hatcheries between May 2003 and February 2004, reported that ceftiofur was used on 76% of the surveyed hatcheries [96]. In 2003, CIPARS reported the percentage of *S.* Heidelberg from retail chicken resistant to ceftiofur/ceftriaxone at 65% and 16% in Québec and Ontario, respectively [97]. These data led to a voluntary cessation of ceftiofur use in hatcheries in Québec. Subsequently, the frequency of CSH from retail chicken in Québec decreased to 7% in 2006 [97]. However, ceftiofur use resumed in 2007, with consequent increases of resistance (Fig. 2).

CIPARS surveillance of sentinel broiler chicken farms commenced in 2013, with 31% of the associated hatcheries reporting ceftiofur use [39]. CIPARS surveillance of sentinel turkey farms also commenced in 2013, expanding from one to three provinces from 2013 to 2016. In 2013, 3% of the 29 turkey flocks reported ceftiofur use [13]. During 2013–2014 ceftiofur resistance among *S.* Heidelberg increased to 58% in retail chicken and 29% in retail turkey [27, 39].

In May 2014, the Canadian poultry industry banned the preventive use of Category I antimicrobials, including ceftiofur, in commercial meat birds (chicken and turkey), and for layers and breeders [98, 99]. Since 2014, ceftiofur has not been reported to be used by turkey producers, and since 2015 there has been no reported ceftiofur use by chicken producers [13, 28]. Information on AMU practices in broiler breeders is currently a data gap; CIPARS has a research project underway to address this data gap.

At the national manufacturer/distributor level, the quantities of antimicrobials reported to CIPARS by the Canadian Animal Health Institute are not stratified by type of cephalosporin, nor are they historically separated by animal species. Consequently, temporal trends are not specific to ceftiofur or to use in poultry. The quantities of cephalosporins distributed since 2014 have remained relatively stable [13].

##### Information on the relationship between the use of the antimicrobial agent(s) and the occurrence of resistant microorganisms or resistance determinants in the food commodity of concern

Figure 2 demonstrates how the frequency of ceftiofur resistance in *S.* Heidelberg from various sources followed changes in ceftiofur use practices.

#### Human uses of the antimicrobial agent(s)

##### Spectrum of activity and indications of treatment

3GCs, such as ceftriaxone, cefixime, cefotaxime and ceftazidime, are authorised for humans in Canada. They are mostly administered parenterally, although some are available in formulations for oral administration.

No single 3GC is ideal for every indication, due to differences in activity and pharmacokinetic features, but they are indicated for a wide range of bacterial infections involving the respiratory tract, urinary tract, skin and soft tissues, intra-abdominal organs and the central nervous system [100].

According to the World Health Organization's list of essential medicines, cefixime is a second line treatment choice for acute invasive bacterial diarrhoea/dysentery, as well as for infections caused by *Neisseria gonorrhoeae*, whereas ceftazidime should only be used for World Health Organization designated priority diseases needing specialised management [101]. Cefotaxime and ceftriaxone are both considered first line treatments for acute bacterial meningitis, severe community acquired pneumonia, complicated intra-abdominal infections, nosocomial pneumonia and severe prostatitis or pyelonephritis, as well as second line treatment choices for bone and joint infections, mild to moderate prostatitis or pyelonephritis and sepsis in paediatric patients [101]. In addition, ceftriaxone is considered first line treatment for *N. gonorrhoeae* and second line treatment for acute invasive bacterial diarrhoea/dysentery [101]. These indications are similar to what ceftriaxone is licensed for in Canada [102].

In North America, empirical treatment for *Salmonella*, when indicated and whilst waiting for susceptibility testing, includes the 3GCs, fluoroquinolones and macrolides [12, 103]. It is recommended that treatment for life-threatening infections should include both a quinolone and a 3GC, until susceptibilities are known and focused treatment can be initiated [103]. In children and pregnant women, treatment with quinolones is relatively contra-indicated, limiting treatment options to 3GC [12]. Ceftriaxone is the treatment of choice for *Salmonella* infection of the meninges, the central nervous system or infective endocarditis, due to its ability to penetrate the blood–brain barrier and cardiac vegetations and devitalised tissue [104].

##### Importance of the antimicrobial agents including consideration of critically important antimicrobial lists

See information in ‘Description of the AMR food safety issue’. Additionally, in 2017, the World Health Organization revised its Essential Medicine list, adding three new categories for antimicrobials: Access, Watch and Reserve [101]. The 3GCs (cefixime, ceftriaxone, cefotaxime and ceftazidime) are in the ‘Watch’ category due to their higher resistance potential, and are only to be utilised for very specific indications [101].

##### Distribution, cost and availability

All costs are indicated in Canadian dollars per 1000 inhabitant-years ($/1000 inh-years) and rates of AMU are expressed as the number of defined daily doses per 1000 inhabitant-years (DDDs/1000 inh-years).

In Canada, public funding of antimicrobials is regulated at the provincial level. All provinces fully fund cephalosporins used in hospitals for in-patient treatment, however, funding differs between provinces when antimicrobials are dispensed by community pharmacies for outpatient treatment. For example, cefixime is funded without restrictions by Ontario and Alberta when dispensed by community pharmacies [105, 106], but British Columbia, Manitoba and Saskatchewan only fund it when appropriate additional information is provided [107–109]. All provinces had similar costs per unit of cefixime in liquid ($0.43–$0.47/ml) and tablet ($2.72–$3.40/tablet) formats, as well as for parenteral ceftriaxone ($12.5–13.5/g active ingredient) [105–109].

Hospital and community pharmacy expenditure associated with 3GC purchasing and dispensing in Canada has varied from year to year [110], and does not necessarily reflect use trends, as the drivers that influence cost do not necessarily influence use.

In 2010, hospital purchases accounted for 73% of all ceftriaxone expenditure; by 2016 this proportion has dropped to 57%, indicating that the costs associated with ceftriaxone purchasing and dispensing have shifted towards dispensing in communities [110]. The reason for this shift is unclear.

##### Availability of alternative antimicrobial agents

Alternatives for 3GCs are available in North America. Alternative treatment choices are directed by the site of infection, causative bacteria, susceptibility testing, patient hypersensitivity and severity of infection. The Infectious Diseases Society of America recommends ciprofloxacin, trimethoprim-sulfamethoxazole or amoxicillin (when antimicrobial treatment is indicated) as treatment alternatives for gastroenteritis caused by NTS [12]. Other alternatives, especially for extra-intestinal infections, include monobactams (aztreonam) and carbapenems [12], both of which are considered critically important for human medicine [8]. Carbapenems may be the last drug of choice for invasive *Salmonella* infections that are resistant to ciprofloxacin and ceftriaxone [111].

##### Trends in the use of antimicrobial agent(s) in humans and information on emerging diseases due to microorganism(s) resistant to the antimicrobial agent(s) or classes

**Fig. 3.**
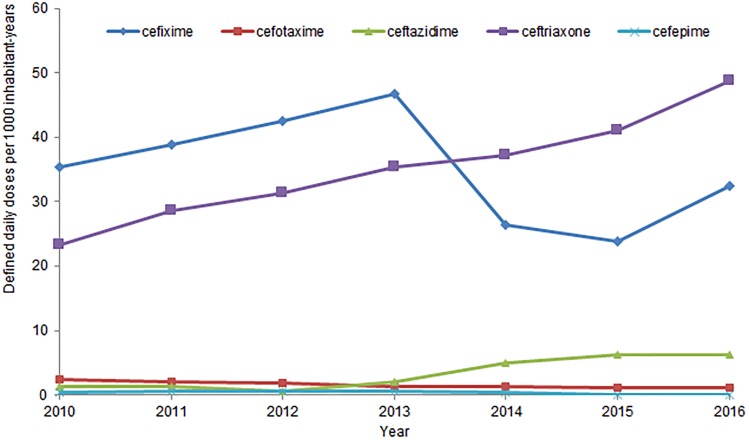
Human consumption of 3rd and 4th generation cephalosporins, from 2010 to 2016, in Canada.

In Canada, human antimicrobial consumption data is compiled by IQVIA which provides community pharmacy dispensing and hospital purchasing data [112]. The trends in consumption of 3GC dispensed by community pharmacies and purchased by hospitals, expressed as DDDs/1000 inh-years, from 2010 to 2016, are presented in Figure 3. For comparison, data for cefepime, a 4^th^ generation cephalosporin, are also included. The annual rate of consumption of 3^rd^ and 4^th^ generation cephalosporins in Canada has increased by 41% from 2010 to 2016 [110]. Between 2010 and 2016, ceftriaxone consumption increased by 113%, from 23 to 49 DDDs/1000 inh-years [110].

In 2016, 3^rd^ and 4^th^ generation cephalosporins were, like the previous 6 years, the class of antimicrobials most purchased by Canadian hospitals, at a rate of 138 DDDs/1000 inh-years [112]. This is 73% more than the second most purchased class of antimicrobial, fluoroquinolones. [112]. Ceftriaxone was the most purchased cephalosporin, and the 5^th^ most purchased antimicrobial overall, at 40 DDDs/1000 inh-years, across all Canadian hospitals [112]. No newly emerging diseases due to CSH have been identified.

#### Summary level of concern and data quality

The level of concern in this section is considered a three; as ceftiofur use in poultry has been observed to temporally mirror trends in ceftiofur resistance in poultry, and the high and increasing levels of ceftriaxone use in humans are indicative of this drug's importance in human medicine in Canada. The data quality for ‘Information on the antimicrobial agent(s) to which resistance is expressed’ is 11, as recent Canadian information from active surveillance is available to describe the AMU of the antimicrobials of interest in both animals and humans. There is, however, a data gap with respect to ceftiofur use in breeder flocks.

### Information on the food commodity(ies)

#### Sources (domestic and imported), production volume, distribution and per capita consumption of foods or raw material identified with the AMR hazard(s) of concern

Poultry production in Canada is a large and economically important industry. In 2016, there were 2817 chicken and 551 turkey producers, which produced 1.2 billion kg of chicken and 183 million kg of turkey meat [113, 114]. The national chicken production volume has almost doubled in the past two decades [113].

In 2016, Canada imported a total of 164.6 million kg of chicken meat and chicken meat products (mostly from the USA and Brazil) and 7.8 million kg of turkey meat and turkey meat products [114, 115]. During the same year, 123 million kg of chicken meat/chicken meat products and 25 million kg of turkey meat and turkey meat products were exported [115]. Canadian poultry products are exported to 67 countries, with the two largest importers being the USA and the Philippines [115].

Pursuant to the North American Free Trade Agreement (NAFTA), the combined import of broiler hatching chicks and eggs is 21% of the current (anticipated) year's production [116]. For chicken, imports under NAFTA are 7.5% of previous year's production, and for turkeys, the volume of import is set annually at 3.5% of domestic production [116].

Canadian consumption of poultry meat has increased steadily over the past two decades. In 2016, Canadians consumed 32.5 kg of chicken and 4.3 kg turkey meat per person, compared to 19 kg beef and 17 kg pork per person [25, 114].

##### Characteristics of the food product(s) that may impact risk management (e.g. further processed, consumed cooked, pH and water activity)

Chicken and turkey can be purchased fresh/chilled, frozen or cooked; which can affect consumer exposure to the foodborne pathogen. From 2011 to 2014, CIPARS conducted a pilot study of retail breaded chicken products (nuggets, strips etc.). Of all the *Salmonella* isolated (*n* = 494), 32% were *S.* Heidelberg, of which 43% were resistant to ceftiofur/ceftriaxone. Frozen raw breaded chicken products can appear cooked when they are in fact raw. This may affect consumer behaviour, leading to an increased risk of exposure to CSH. A Canadian case-control study found that one third of the study participants did not handle or prepare raw chicken strips or nuggets with the same level of caution as they would do with raw chicken [44]. Thirty percent of participants reported washing their hands less often after handling processed products, in comparison with handling raw whole chicken, due to a perception that these products had already been cooked, and was, therefore, safer [44]. For context, raw chicken (including frozen breaded chicken) has been a source of 18 *Salmonella* outbreaks in Canada since 2017 [117].

##### Description of the food production to consumption continuum (e.g. primary production, processing, storage, handling, distribution and consumption) and the risk factors that affect the microbiological safety of the food product of concern

The information in this section is presented in the context of the production process, including farm, transport, processing (stun and killing, scalding, de-feathering, evisceration, washing, chilling and portioning), product storage (chilled or frozen) and retail and consumer handling.

Chicken and turkey production in Canada is supply managed. Canadian chicken production involves importing male and female broiler breeder lines (as either hatching eggs or chicks) from the USA; American companies own the elite stocks (e.g. pure line, great grandparents and grandparents; Supplementary Fig. S1). In 2016, Canada imported ~2.8 million broiler breeder hatching eggs, which were hatched in domestic hatcheries and 3.2 million broiler breeder chicks (hatched and processed in American facilities) [118]. Commercial broiler farms imported 127.5 million hatching eggs and 20.8 million chicks during the same year [118]. Turkey primary breeding stock (great grandparents and grandparents) is largely situated in Canada.

The hatcheries, regulated by the Canadian Poultry and Egg Processors Council, provide standard day-old-chicks, from both domestic and imported sources, to commercial broiler producers. At the hatchery, the eggs are vaccinated against Marek's disease and ceftiofur (where used) is administered, before they are placed in a hatcher. In hatcheries that do not utilise *in-ovo* vaccination, the Marek's Disease vaccine and/or the antimicrobial are given subcutaneously at one day of age. At one day of age, they undergo processing (additional vaccinations against infectious bronchitis and/or coccidiosis, health check, and, in the case of turkey poults, gender identification), and are placed in boxes for shipping to farms for rearing. A 42 g chick (Ross 308 standard) could reach 2.1 kg in 36 days. In Canada, turkeys reach market weight at between 11 and 17 weeks, and are produced in various weight ranges, including broiler turkeys (up to 6.2 kg), hen turkeys (6.2–10.8 kg) and tom turkeys (⩾10.8 kg) [119].

Apart from antimicrobial selection pressure, there are multiple factors at hatchery or farm level that can influence the colonisation, prevalence and growth of *Salmonella*, or the dissemination of resistance determinants. Possible sources of *Salmonella* contamination include water, feed, litter, staff and the environment [120]. Risk factors include high stocking densities, vertical integration, contamination of a previously placed flock, inadequate hygiene and contaminated day-old chicks from hatchery [82].

Resistant bacteria could be introduced by mixing medicated and un-medicated chicks in one barn (i.e. when there are importation and inter-provincial exchanges of chicks) [121] or disseminated from earlier levels of production such as grandparent flocks to their broiler progenies [95]. There is evidence that cefotaxime-resistant *E. coli* are persistent from grandparent to broilers, indicating that vertical transfer of resistant strains could occur [95]. In Denmark, research has found *bla*_CMY−2_ positive *E. coli* collected from parent flocks, broilers and chicken meat had identical genetic fingerprint patterns, suggesting that resistant *E. coli* genotypes persist throughout broiler processing, exposing consumers to resistant bacteria through chicken meat [15]. Cephalosporins have never been approved for use in poultry in Denmark; the presence of extended-spectrum cephalosporin-resistant *E. coli* in poultry may be due to vertical transmission from imported grandparent stock from another country where cephalosporins were used [15].

Poultry manure often harbours resistant microbes and could be spread on a field used for feed or food crop production, which may affect the dissemination of resistant microbes, including CSH [122, 123]. The possibility that poultry feed could be contaminated with *S*. Heidelberg exists. However, in 2016, Canadian feed sampling did not identify any *S.* Heidelberg among the 46 *Salmonella* isolated from feed [13]. Furthermore, none of the *Salmonella* isolates were resistant to *β*-lactams [13].

No after-farm activities were identified which would induce preferential selection of different serovars or resistant strains, although some may impact pathogen load. It is not anticipated that transport or the stunning and killing process would result in increased growth or survivability of CSH in comparison with susceptible *S*. Heidelberg strains [124].

Cross-contamination during abattoir processing may occur at any stage, but is generally associated with increased handling and contact between carcasses (or their fluids) during de-feathering, evisceration, washing and immersion (water) chilling. Following slaughter, carcasses are immersed in a scalding vat to aid de-feathering. Scalding is generally associated with a net decrease in microbial contamination, due to high temperatures capable of *Salmonella* inactivation, and a wash-off effect. *D*-Values of 1.1–5.9 min at scald temperatures of 55 °C, both in scald water and on broiler skin, were reported [62]. De-feathering is associated with low levels of cross-contamination of nearby carcasses and surfaces [125]. The net effect is a reduction in the variability of microbial loads between the carcasses within a flock.

When viscera are removed without damage, evisceration is associated with a net decrease in microbial contamination. However, viscera may rupture during removal, contaminating the carcass. When gross contamination is detected, carcasses are condemned, but processing equipment may remain contaminated, disseminating CSH to other carcasses.

Regarding the effect of portioning, assuming bacteria are distributed uniformly across the carcass, the bacterial load on individual portions may be modelled as a function of the portions' relative masses or surface areas. More complex models assume the occurrence of bacterial clustering on specific cuts; the degree to which this clustering occurs is a best-guess estimate [125, 126].

Consumer behaviour, in particular, unsafe food handling and preparation, is a critical risk factor for increasing the probability of exposure to *S*. Heidelberg (susceptible or resistant to ceftiofur/ceftriaxone). Further discussion on risk management options at the consumer-level can be found in ‘Measures to minimise the contamination and cross-contamination of food by resistant microorganism(s)'.

#### Summary level of concern and data quality

The level of concern is estimated a three, as poultry meat is commonly consumed, and there are various pressure points during the production stages on a farm where AMU can select resistance, or where CSH can be disseminated (also later in the food chain). The data quality score for ‘Information on the Food Commodity(ies)’ is 8; the rationale being that although recent Canadian data are available, the poultry production information is empirical.

### Information on adverse public health effects

#### Characteristics of the disease caused by the identified foodborne resistant microorganisms or by pathogens that have acquired resistance determinants via food

##### Trends in AMR foodborne disease

Between 2003 and 2011, 638 cases of invasive *S.* Heidelberg infections were reported in Canada, with 30% occurring in children under the age of 13 years [127]. During 2016, *S.* Heidelberg accounted for 7% of all reported human salmonellosis cases in Canada [16]. The reported 2016 incidence of *S.* Heidelberg isolated from humans (1.6/100 000 individuals) represents a 13% decrease from the average incidence observed from 2007 to 2011 (1.8/100 000) [16]. However, this does not account for potential under-reporting, for example, in Ontario, it is estimated that for every *Salmonella* case reported, between 13 and 37 cases go unreported [128].

The percentage of human *S*. Heidelberg isolates resistant to ceftiofur/ceftriaxone has varied over time; from 2007 to 2010 resistance was 14–19%, between 2011 and 2015 it increased to 27–33%, and in 2016, it decreased to 16% [13].

A quantitative stochastic risk model was developed to estimate the number of human infections caused by CSH in Ontario and Québec, which considered changes in AMU practices in chicken production over time, the under-reporting of *Salmonella* cases and uncertainty in the data provided [127]. The results demonstrated that changes in the incidence of CSH in people paralleled the changes in AMU practices in the poultry industry, in line with a previous finding of a significant temporal correlation between ceftiofur resistance in *S*. Heidelberg isolated from retail chicken and from humans [127, 97].

##### Frequency and severity of effects including case-fatality rate, hospitalisation rate and long-term complications

The invasive nature of *S.* Heidelberg infection increases the risk of severe and life-threatening illness, compared to other *Salmonella* serovars. It is estimated that bacteremia develops in 5–8% of people infected with NTS [104]. The addition of resistance to this already virulent microorganism will likely increase morbidity.

Currently, no national burdens of illness data, such as the number of *S.* Heidelberg cases hospitalised or the incidence of treatment failures due to resistance, are routinely collected in Canada. A Canadian *S.* Heidelberg case-control study (not specific to CSH), conducted in 2003 as a result of an outbreak investigation, found that the median length of illness was 10 days, 47% of the cases were admitted to hospital with a 5-day median length of hospitalisation, and one third of the cases had bloody diarrhoea [44].

From 1996 to 1999, *S.* Heidelberg was estimated to have caused 84 000 cases of illness (6% of all salmonellosis cases) annually in the USA, with 11% of the infections being invasive in nature [48, 129]. During this timeframe, *S.* Heidelberg was isolated from 7% of the patients who died from salmonellosis, second only to *S.* Typhimurium (50%) [130].

An *S*. Heidelberg outbreak in the USA between March 2013 and July 2014, linked to consumption of chicken from one farm, resulted in 634 human cases, with a 38% hospitalisation rate, and no mortalities [131]. The AMR profile of these isolates was multi-class resistant, but did not include ceftriaxone resistance [131].

The CDC published a report on AMR threats in the US, associating drug-resistant NTS with ~1.2 million illnesses, 23 000 hospitalisations and 450 deaths annually [2]. The AMR patterns of concern noted in the report included resistance to ceftriaxone (36 000 illnesses and 13 deaths per year) [2]. In Canada, in 2014, 31% and 37% of the *S.* Heidelberg isolated from blood and urine, respectively, were resistant to ceftriaxone. In 2015, 95% of *S.* Heidelberg from blood and 93% from urine were resistant to ceftriaxone. Data from 2016 indicated a decrease in the percentage of *S*. Heidelberg isolates resistant to ceftriaxone, with 18% from blood, and 15% from urine resistant to ceftriaxone.

##### Susceptible populations and risk factors

The following description of susceptible populations and risk factors is generic for all *Salmonella*. No additional risk factors specifically for *S.* Heidelberg or CSH were found. Extremes of age, immunodeficiency, prior AMU, neoplastic disease, gastric hypoacidity, pernicious anaemia, diabetes, rheumatological disorders, recent bowel surgery and malnutrition can all increase susceptibility to *Salmonella* infection [104, 132]. Kidney stones, gallstones, atherosclerotic endovascular lesions, schistosomiasis and prosthetic devices may provide sites for prolonged *Salmonella* infection [104]. Due to relatively lower stomach acidity, and the buffering effect of milk, neonates are particularly at risk, whereas the elderly, especially those in nursing homes, are more susceptible due to chronic co-morbidities and weakened immune systems [132]. For those ⩾50 years old, the case-fatality rate is estimated to be 1% higher than their younger counterparts [132]. Risk factors associated with a higher probability of vascular infections due to NTS include the male sex, hypertension and coronary arterial disease [133].

##### Epidemiological pattern (outbreak or sporadic)

Like other *Salmonella*, *S.* Heidelberg has been implicated in both outbreaks and sporadic infections [134–136]. Various multistate outbreaks of *S.* Heidelberg in the USA have been linked to a single poultry producer [136], to the consumption of ground turkey [137], and to contact with dairy calves [138]. A Canadian case-control study demonstrated that 34% of *S.* Heidelberg infections were attributable to home-prepared chicken nuggets and strips [44].

##### Regional, seasonal and ethnic differences in the incidence of foodborne disease due to the resistance hazard

Other than the noted difference between the provinces in the frequency of *S*. Heidelberg isolates resistant to ceftiofur/ceftriaxone, the available data did not identify any other regional, seasonal or ethnic differences. However, this may be because these elements were not requested during the primary data collection.

##### Additional information on the relationship between the presence of the resistant microorganisms or determinants in the food commodity and the occurrence of the adverse health effect(s) in humans

No additional information was available.

##### Consequences of AMR on the outcome of the disease: increased frequency and severity of infections, including prolonged duration of disease, loss of treatment options and treatment failures, increased frequency of bloodstream infections, hospitalisation and mortality

The consequences of infections caused by resistant bacteria encompass all aspects of morbidity, management and outcomes of such infections. These aspects are difficult to separate, and as such, we combined two Codex subheadings (Loss of treatment options and treatment failures and Increased frequency and severity of infections, including prolonged duration of disease, increased frequency of bloodstream infections, hospitalisation and mortality) into the heading ‘Consequences of AMR on the outcome of the disease: increased frequency and severity of infections, including prolonged duration of disease, loss of treatment options and treatment failures, increased frequency of bloodstream infections, hospitalisation and mortality’.

Examples of the burden of illness measures associated with resistant *Salmonella* reported in the literature can be found in Supplementary Material Table S4. Bacteremia [139–142] and hospitalisation [139, 140, 143–149] were significantly more common among patients infected with resistant *Salmonella*, and hospital admissions tended to be longer in duration [139, 140, 150]. Depending on how many antimicrobial classes an isolate was resistant to, the risk of having a bloodstream infection was 3–10 times higher, risk for hospitalisation was up to 4 times higher, and the risk of staying in hospital longer than 3 days were up to twice as high, compared to infection by susceptible strains [140]. These resistant infections were also associated with increased morbidity [151, 152], increased mortality rates [143–146, 148, 151, 153] as well as loss of productivity and increased hospital costs [150, 154]. It is notable that the only *S.* Heidelberg specific outbreak with published burden of illness information was a 1987 hospital outbreak in England [154]. In this particular study, the microorganism was described as ‘multiply-resistant’ *S.* Heidelberg, but no details of the resistance pattern were provided [154].

#### Summary level of concern and data quality

Loss of treatment options/treatment failures due to CSH infections is possible. The lack of information on the burden of illness specific to 3GC-resistant *S.* Heidelberg constitutes a large data gap. The level of concern was estimated at three, based on the availability of sufficient information to confirm CSH's association with worse disease outcomes. The overall data quality for ‘Characteristics of the disease caused by the identified foodborne resistant microorganisms or by pathogens that have acquired resistance determinants via food’ is 11 (Canadian data, passive surveillance from 2015 onwards) and 6 for ‘Consequences of AMR on the outcome of the disease: Increased frequency and severity of infections, including prolonged duration of disease, loss of treatment options and treatment failures, increased frequency of bloodstream infections, hospitalisation and mortality’ (due to the lack of recent Canadian specific data from active or passive surveillance). The overall average data quality score for ‘Information on adverse public health effects’ is 8.5.

### Risk management information

#### Identification of risk management options to control the AMR hazard along the production to consumption continuum, both in the pre-harvest and post-harvest stages

Risk management options can be divided into two additional categories (in addition to the Codex pre-harvest and post-harvest): (1) controlling selection pressure and (2) controlling the spread of resistant bacteria.

##### Measures to reduce the risk related to the selection and dissemination of foodborne antimicrobial-resistant microorganism(s)

The major risk factor for the selection of resistant bacteria is AMU. Measures limiting AMU are likely to be less effective if the need for antimicrobials is not also addressed, e.g. farming practices that reduce the incidence of diseases that require AMU. The European Food Safety Authority has stated, ‘Prioritisation is complex, but it is considered that a highly effective control option would be to stop all uses of cephalosporins/systemically active 3rd/4th generation cephalosporins, or to restrict their use (use only allowed under specific circumstances). As co-resistance is an important issue, it is also of high priority to decrease the total antimicrobial use in animal production in the EU’ [155].

For AMU decisions, it is important to involve every level of poultry production, and to involve multiple stakeholders, such as veterinarians, the pharmaceutical industry, the poultry production industry (producers and industry bodies) and regulatory authorities. The lack of approved alternative antimicrobials with a short drug (residue) withdrawal time, continued disease pressures on poultry flocks, and the continuous pressure on veterinarians to maintain both the health and productivity of a flock need to be understood and addressed.

Options to prevent, limit or decrease AMU may include assessing possible human health impacts of a particular antimicrobial before it is licensed for use in animal food production, removing financial incentives linked to prescribing antimicrobials and the limitation of certain antimicrobials for some indications or in certain food animal species [156]. Other options include the development and implementation of antimicrobial stewardship programs aimed at stakeholders at all levels, as well as addressing prescriber behaviour (e.g. placing restrictions on the prescriptions of specific antimicrobials, continued prescriber education, auditing and improved diagnostic services to direct AMU) [156]. Publishing and disseminating up-to-date prudent use guidelines should be ensured. Current antimicrobial prudent use guidelines produced by the Canadian Veterinary Medical Association (2008) state that for poultry and *E. coli*, in the ‘*case of recurring omphalitis related to a breeder flock or poor shell quality in times of very hot weather, the use of antibiotics in the hatchery* is *judicious*’ [157], recommending that gentamicin and lincomycin–spectinomycin be used (i.e. ELU). Canadian AMU and AMR surveillance data should be taken into consideration to update these guidelines (see ‘Effectiveness of current management practices in place based on surveillance data or other sources of information’).

##### Measures to minimise the contamination and cross-contamination of food by resistant microorganism(s)

At the farm-level, interventions can be targeted at preventing introduction and spread of *Salmonella*; including the implementation of specific *Salmonella* control programs at the breeder/parent stock, hatchery and broiler farm level, and addressing all possible sources of exposure (water, feed, litter, farm staff and environment) [120]. According to the World Health Organisation and the Food and Agriculture Organisation of the United Nations, the four most important control measures during primary production are: (1) eliminating *Salmonella* from grandparent and parent stocks, (2) having an all-in all-out production system at the broiler farm, (3) avoidance of pathogen transfer between flocks by contaminated processing equipment through logistic slaughter planning and (4) transport crate cleaning [120].

Biosecurity is also of particular importance, and should encompass all aspects of farm/barn access (people and vehicles), pest control (rodents, insects and wildlife), water and feed management, clothing and footwear of staff, sufficient decontamination procedures and proper protocols for quarantine and the handling of sick/dead birds.

Interventions during processing are designed to reduce cross-contamination between carcasses, and to physically remove or chemically inactivate *Salmonella*. A few measures are highlighted, but it is not an exhaustive list of all possible risk management options.

Logistic slaughter is a risk management measure, where potentially contaminated flocks are processed after those unlikely to be contaminated [120]. Other measures include high-scalding temperatures, maintaining and decontaminating equipment and prevention of viscera rupture during evisceration [120]. Washing of carcasses can include sequential washing, with wash additives, such as acidified sodium chlorite, citric acid or sodium hypochlorite [120]. Acidified sodium chlorite (pH ~ 2.5) at concentrations of 700–900 ppm has been associated with decreased *Salmonella* prevalence of 18–56% [120]. Chilling (air or immersion) should be rapid and chemicals, such as chlorine or other chlorine derivatives, may be added to the chilling water [120].

Temperature control during packing, storage and at point of sale (maintaining cold chain) is important. Salt concentration and pH can affect bacterial growth in retail meat products. For *S.* Heidelberg, 8% or 10% salt concentration at pH 6.8 inhibited all bacterial growth at all temperatures tested, but at 6% salt and 20–35 °C, growth occurred within 1 week [158].

At the consumer level, education and awareness campaigns may be implemented to promote safe food handling and cooking practices. Cooking is a critical control point for protecting human health through the inactivation of *Salmonella* in food. The *D*-values reported for *Salmonella* in meat products typically range between 1 and 10 min at 60 °C and less than 0.1 min at 70 °C [159]; high-fat content or low-water activity increase the time required to inactivate the organism. A 7 log_10_ reduction of *Salmonella* can be obtained by cooking poultry meat to a minimum internal temperature of 74 °C [120].

A Canadian *S.* Heidelberg case-control study found that 23% of participants reported that children and teenagers were typically the consumers of frozen processed chicken products, products that are associated with an increased risk of transmitting *Salmonella*, while 40% reported that the whole family consumed the products [44]. Notably, 12% of the participants never read the cooking instructions for these products, representing a potential risk management intervention point [44]. On July 12, 2018, the Canadian Food Inspection Agency issued a notice to industry to implement control measures by April 1, 2019 to reduce *Salmonella* to below detectable amounts in frozen raw breaded chicken products [160]. To this end, four options have been proposed: (1) implement a cooking process to achieve a 7-log reduction in *Salmonella*, (2) implement a *Salmonella* testing program for the chicken mixture (to ensure the absence of *Salmonella*), (3) implement a hold-and-test program or (4) implement processes to achieve a 2-log reduction in *Salmonella* and *Salmonella* sampling program [160].

Risk management options at different stages of poultry production, and the relevant parties involved in implementation are described in Table 1.

**Table 1.** Pre- and post-harvest options for controlling ceftiofur/ceftriaxone-resistant *Salmonella* Heidelberg through the poultry production chain

Production stage	Potential intervention	Challenges	Relevant stakeholders
Breeder or parent stock	*Salmonella* control program *Salmonella* vaccines Competitive exclusion products Develop/improve antimicrobial prudent use guidelines Reporting of health status and antimicrobial use information	Require frequent monitoring Monitoring and decontamination costs Lack of current ability to measure effectiveness *Salmonella* vaccines may not decrease the prevalence of *E. coli* or other *Enterobacteriaceae* which might have the same resistance patterns	Drug regulators Producers/producer organisations Research and development professionals Pharmaceutical industry
Hatchery	*Salmonella* control program Develop/improve antimicrobial prudent use guidelines Prohibition/restriction of the use of 3GCs such as ceftiofur Optimal hatching conditions Only setting good quality eggs Reporting of antimicrobial use information	Require frequent monitoring or surveillance Monitoring and decontamination costs Lack of current ability to measure effectiveness May require regulatory decisions	Producers/producer organisations Veterinarians/veterinary associations Food inspection and enforcement authorities Drug regulators
Broiler farm	*Salmonella* control program Broiler chicks hatched from problematic breeder flocks (e.g. high bacterial contamination in older breeder flocks with oophoritis and salpingitis) may be targeted for medication to reduce infections Develop/implement antimicrobial stewardship education programs Improved hygiene/cleaning between batches of broilers Control measures regarding poultry manure disposal and use (e.g. storage, compositing, and fertiliser use)	Require frequent monitoring or surveillance Monitoring and decontamination costs Lack of current ability to measure effectiveness Cost of stewardship programs Shared responsibility among professionals, producers and governments	Producers/producer organisations Veterinarians/veterinary associations/veterinary schools Pharmaceutical industry Government agencies Manure users
Abattoir and meat processing	Logistical slaughter planning Physical/chemical decontamination measures (i.e. type of scalding, washing, type of chilling)	Improve hazard analysis and critical control points (HACCP) procedures	Poultry processing industry Food inspection and enforcement authorities
Storage retail consumer handling	Optimal packing, transport and storage methods Consumer awareness and education regarding safe food handling and preparation procedures	Requires monitoring Cost-effectiveness of monitoring	Processing industry Transport industry Retail industry Consumer groups Public health units Regulatory authority

##### Effectiveness of current management practices in place based on surveillance data or other sources of information

Evaluating the effectiveness of different risk management options is difficult due to the lack of quantitative data of the enumeration of CSH, and *Salmonella* in general, at different points along the production-to-consumption continuum. It was furthermore considered outside the scope of this risk profile (but inside the scope of a future quantitative risk assessment) to comprehensively evaluate the effectiveness of every possible risk management option. Thus, this section focuses primarily on vaccines and AMU.

Since January 2013, all broiler breeder flocks in Ontario have been receiving live attenuated *S*. Typhimurium vaccine, as well as killed autogenous vaccine for *S.* Enteritidis, *S*. Typhimurium, *S.* Heidelberg and *S*. Kentucky [161]. In January 2017, a *Salmonella enterica* subsp. *enterica* serovar Infantis autogenous vaccine was added. For the 5 years before vaccination commenced, the average frequency of *Salmonella* positive samples from hatcheries and breeder barn environments was 10% and 9% respectively, compared to 3% and 4% average for the 4 years after vaccination was implemented [161]. Although is not possible to attribute the decrease in *Salmonella* prevalence directly to vaccinations, the results are highly suggestive [161], and vaccination as a viable alternative to AMU bears further investigation. It has been suggested though that the use of vaccines, and other bacterial limiting interventions, aimed at only *S*. Enteritidis and *S*. Typhimurium may increase selection pressure for other serovars, such as *S*. Heidelberg [82]. The *Salmonella* vaccines registered for use in Canada are predominantly specific for *S*. Enteritidis and *S*. Typhimurium, not to *S*. Heidelberg; hence, there is use of the specific autogenous vaccines in the breeder program [162]. Two products claim to reduce *S*. Heidelberg colonisation [162]. Of the broiler chicken flocks surveyed by CIPARS, none reported *Salmonella* vaccine use from 2013 to 2015, but during 2016, 4% (5/136) reported *Salmonella* vaccine use [13, 27, 28, 39].

There is international concern regarding *Salmonella* resistance to 3GC, and several regulatory agencies and industry bodies have taken measures to address the use of cephalosporins in food animals/poultry [72, 163]. In 2012, the European Medicines Agency revoked poultry indications for all veterinary drugs containing systemically administered 3^rd^ and 4^th^ generation cephalosporins [164], the US Food and Drug Administration prohibited certain ELU of cephalosporins in major food producing species, including poultry [165] and the British Poultry Council voluntarily stopped the use of cephalosporins [166]. The World Organisation for Animal Health recommends that 3^rd^ and 4^th^ generation cephalosporins should not be used as first line treatment unless justified, bacteriological tests should support its use as second line treatment, they should not be used for disease prevention via water or feed administration, and ELU should be limited and reserved for situations where no alternatives are available [167].

In terms of effectiveness of changing AMU in Canada on CSH from poultry, the potentially high levels of ceftiofur use in the early 2000s, followed, respectively, by a sharp reduction in use, an intermittent increase in use, and a complete ban, has been mirrored not only in the percentage of *S*. Heidelberg isolates resistant to ceftiofur/ceftriaxone isolated from various stages during poultry production, but importantly also in humans infected with *S*. Heidelberg (Fig. 2). Surveillance data also indicated a similar change in ceftiofur resistance in generic *E. coli* isolated from retail chicken [32]. This is an important finding as the decrease in ceftiofur resistance rates across two different bacterial species is indicative of a shared selective pressure (i.e. AMU).

The apparent correlation between reductions in ceftiofur use and reduced prevalence of ceftiofur resistance in *Salmonella* or *E. coli* has been demonstrated by other countries. For example, in Japan, the prevalence of extended-spectrum cephalosporin-resistant *Salmonella* in chicken meat was 45% in 2011 [168]. In March 2012, the Japanese poultry industry voluntarily ceased using ceftiofur and subsequently the prevalence of resistant *Salmonella* decreased yearly to 11% in 2015 [168].

In 2006, CIPARS data were used in part by Health Canada to develop resistance-related warning statements for ceftiofur products [169]. These warning statements have recommended that ceftiofur products should only be used to treat individual animals, and advised against ELU. They have also included recommendations regarding the need for pathogen culture and susceptibility testing. These statements were developed within Health Canada's ELU framework, which takes into consideration AMR when conducting both pre- and post-market microbial food safety assessments of antimicrobial drugs [169].

#### Summary level of concern and data quality

The temporal mirroring of ceftiofur use practices and ceftiofur/ceftriaxone resistance in two bacterial species suggests that controlling ceftiofur use was a highly effective risk management intervention. The suggested data elements in this section do not lend themselves to scoring a level of concern. However, these risk management options and the effectiveness of current activities raise the question of whether the current (2016) frequency of ceftiofur resistance in *S*. Heidelberg from retail chicken at 12%, from retail turkey at 22% and 16% of human isolates is low enough to be acceptable from a safety perspective [13], or whether additional risk management interventions are needed. As the ceftiofur use on the sentinel poultry farms is reported to be zero, other non-ceftiofur use interventions might have to be considered. The data quality for ‘Risk Management Information' is scored at 12 as current active Canadian surveillance data are available.

### Evaluation of available information and major knowledge gaps

#### Uncertainty of available information and areas where major gaps of information exist that could hamper risk management activities, including, if warranted, the conduct of a risk assessment

The subheadings 7.1 Uncertainty of available information and 7.2 Areas where major gaps of information exist that could hamper risk management activities, including, if warranted, the conduct of a risk assessment were combined. The Codex Guidelines are not specific on how to describe uncertainty or data gaps. For the purpose of this risk profile each section was summarised qualitatively, highlighting uncertainty of information and data gaps, by section heading (Supplementary Table S5). This table also covers a discussion of whether new data (including molecular information from WGS) would be beneficial to make a risk management decision. A summary of this section is included in the discussion.

## Discussion

The information presented in this article, with the levels of concern and data quality ascribed to appropriate sections, suggest that CSH is not just a hazard, but an AMR risk. Provisional measures were, and are still required to decrease the risk to human health posed by CSH from poultry meat. Ceftiofur-resistant *Salmonella* is increasingly isolated around the world, and CSH is frequently isolated from humans and animals in North America. In Canada, CSH is isolated more frequently from poultry than from other healthy food animal sources tested. Human infections caused by *S*. Heidelberg are associated with higher morbidity and mortality, can affect vulnerable sub-populations (e.g. children), and have limited alternative treatment options. Furthermore, resistance determinants are transmissible to other bacterial species and Canadians have high exposure to poultry meat.

The most significant data gaps include the concentration of CSH throughout the production-to-consumption continuum (pathogen load), Canadian burden of illness measures due to CSH, as well as the genetic relatedness of *S*. Heidelberg isolated from poultry and from humans.

One data element not requested in the Codex Guidelines that should be mentioned is the issue of trade barriers related to resistant pathogens. For example, with the American multistate outbreak of multidrug resistant *S*. Heidelberg in 2013/2014, the news media reported that Mexico banned three American chicken processing plants from exporting their products to Mexico [170]. In relation to the same outbreak, the media reported the outcome of one court case concerning a child who suffered a brain injury because of the *S*. Heidelberg infection; the jury attributed 30% of the fault to the chicken farm, and 70% to the family for their handling and preparation of the food [171]. This type of information does not readily translate into the more health-related suggested data elements for a risk profile, but nonetheless is of significant interest to a policy audience. Also, the finding of extended-spectrum cephalosporin-resistant *S*. Heidelberg in poultry imported from Brazil into Portugal in 2014–2015 [85] and in poultry imported from Brazil into the Netherlands between 1999–2013 [83] in part prompted the recommendation from the European Food Safety Authority for consideration of inclusion of Heidelberg in the Europe's recommended *Salmonella* serovar targets for breeding hens [172].

Ending ELU of ceftiofur, particularly preventive use in poultry, is critical for limiting the emergence and spread of ceftiofur resistance. Importantly, the Canadian poultry industry voluntarily eliminated the preventive use of ceftiofur, a risk management intervention mandatory for all poultry producers in Canada [99]. Vaccination programs for *Salmonella*, including *S.* Heidelberg, and other *Salmonella* control programs might be a successful option as well. These interventions should include strategies to measure and monitor their effectiveness.

WGS is expected to be an invaluable tool for increasing our knowledge of *S.* Heidelberg and other foodborne resistant microorganisms. WGS data will allow the mapping of genetic resistance determinants and their transmission pathways, both vertical and horizontal, along the production-to-consumption continuum, as well as the determination and quantification of source attribution in human cases of salmonellosis. In addition, WGS analyses of *S.* Heidelberg from both human and poultry sources in Canada suggest horizontal plasmid dissemination of *bla*_*CMY−2*_ containing IncI1 plasmids among *S.* Heidelberg isolates, rather than just clonal spread [58]. To date, WGS has shown its discriminatory power for differentiating outbreak and sporadic *S.* Heidelberg strains, which was not possible using the conventional pulsed-field gel electrophoresis typing method due to its clonality [173–175]. Additional Canadian WGS work is underway to examine the spatial and temporal distribution of CSH, and to evaluate the potential value of integrating WGS data into a production-to-consumption quantitative microbial risk assessment, using WGS data from *S.* Heidelberg collected in Canada [176]. WGS is valuable in hazard characterisation by identifying the presence and location of AMR genes, and by determining the virulence profile of the bacteria of interest [176]. In addition, WGS enables better measure of risk of exposure and, once correlations between genotypic and phenotypic profiles are validated, should enable better predictions of clinical outcomes [176]. A recent US study highlighted the value of WGS in examining the impact of exogenous farming practices on the virulence, AMR and survival of *S.* Heidelberg [81].

Regarding the Codex Guidelines, Appendix A of the Codex *Guidelines for Risk Analysis of Foodborne Antimicrobial Resistance* provided an excellent template for compiling a risk profile with relevant information in a structured, transparent manner. However, compiling the risk profile was challenging, and some aspects may need refining. For instance, guidance on how to objectively derive conclusions from the data was lacking. All the suggested elements for inclusion in the risk profile are currently weighted equally. However, specific elements or findings may have more importance in some situations, based on trade, health, or policy considerations. Challenging aspects also included determining when to cease adding information from other animal species, other bacterial species, other countries or other related antimicrobial agents to inform the issue. Condensing the material into a suitable length with adequate non-technical language for policy makers was also problematic. Future versions of Codex AMR risk profiles may consider including slightly different content to reflect the need for conciseness and to narrow the scope of material for specific sections.

Some headings in the Codex Guidelines lacked clarity on the information that was needed, or alternatively resulted in duplication of information. For example, there are sections for the animal and human uses of the antimicrobial which refer to cost of the antimicrobial of interest, but it is unclear if cost, in this context, refers to the price of the antimicrobial to the consumer, or to expenditure associated with its use. In addition, the value of providing cost information, and its interpretation within the context of a risk profile, is undefined. The section on ‘Availability of alternative antimicrobial agents' does not clearly indicate if alternative antimicrobial treatment options refer to only infections caused by the AMR hazard of interest, or if the information provided should encompass a broader discussion of treatment alternatives in different scenarios, e.g. in patients that are hypersensitive to cephalosporins.

‘Growth and survivability, including inactivation in foods (*D*-value, minimum pH for growth) of the foodborne antimicrobial-resistant microorganism(s) in the food commodity production-to-consumption continuum’, ‘Description of the food production to consumption continuum (e.g. primary production, processing, storage, handling, distribution and consumption) and the risk factors that affect the microbiological safety of the food product of concern’ and ‘Measures to minimise the contamination and cross-contamination of food by resistant microorganism(s)’ are duplicative, and combining them into one heading would yield a more concise delivery of the material. The headings for ‘Trends in the use of the antimicrobial agent(s) in the agricultural and aquaculture sectors and information on emerging resistance in the food supply’, ‘Information on the relationship between the use of the antimicrobial agent(s) and the occurrence of resistant microorganisms or resistance determinants in the food commodity of concern', and ‘Trends in the use of antimicrobial agents(s) in humans and information on emerging diseases due to microorganism(s) resistant to the antimicrobial agent(s) or classes' also result in duplication of material and a revision of these headings may provide some clarification.

To improve the order of presentation of information, ‘Information on the antimicrobial agent(s) to which resistance is expressed’ could be placed before ‘Information on the antimicrobial resistant organism and/or AMR determinant’. Discussion and interpretation of AMR requires knowledge of the antimicrobial of interest.

The Codex Guidelines are not specific on how to describe uncertainty or data gaps. For the purpose of this risk profile, uncertainty and data gaps in each section were summarised qualitatively and transparently, however improvements or standardisation of this in a streamlined manner (to not unduly impact the timeliness of the risk profile process) are needed.

Compiling the risk profile was time and human resource intensive. Dedicated staff to create a risk profile would be preferential to ensure timely completion, which would optimistically, take ~6 months to complete. As more risk profiles are created, completion time may shorten, as information may be applicable to more than one risk profile. For example, a risk profile for another pathogen in poultry (e.g. *Campylobacter* spp.) would have some identical elements to this risk profile, such as poultry industry information, or per capita chicken consumption data. Sections of the risk profile also require specific expertise. Thus, to compile an accurate risk profile, personnel with expertise from a variety of scientific fields were needed. Authors of this risk profile included veterinarians, microbiologists, epidemiologists, physicians and public health experts, amongst others.

It is important to note that while a risk profile is not intended to be an abbreviated risk assessment, our work evolved into being a qualitative risk assessment. Ensuring that clear and concise questions are addressed by a risk profile is a key recommendation from our experience with implementing the Codex Guidelines. With refining and clearer guidance on the depth and breadth of the required information presented in each section, these guidelines will be an even more valuable tool in describing a food safety problem and its context.

Risk profiles are intended to lead to recommendations either to take further action, prompt a foodborne AMR risk assessment, establish additional information gathering mechanisms and/or implement immediate risk mitigating measures. Overall for this risk profile, we recommend that further action is needed, as the current level of CSH in humans is still above 16%, despite some very effective risk management interventions. We recommend that the three major data gaps (pathogen load, burden of illness and genetic similarity of CSH between poultry and people) be addressed. In addition, we recommend that to quantitatively evaluate the success of current and future risk management interventions, a quantitative microbial risk assessment be conducted. Finally, as a risk management tool, risk communication per the Codex Guidelines [7] can be utilised to communicate the outcomes of this risk profile with the stakeholders for collaborative efforts to tackle the AMR food safety issue.

## Supplementary material

For supplementary material accompanying this paper visit https://doi.org/10.1017/S0950268819001778. click here to view supplementary material
