# Supplementary material for: Ceftiofur-resistant Salmonella enterica serovar Heidelberg of poultry origin – a risk profile using the Codex framework
Source: Epidemiol Infect. 2019 Nov 4;147:e296. doi: 10.1017/S0950268819001778 (PMC6836576; doi:10.1017/S0950268819001778)
Supplement: Supplementary file 1 [file S0950268819001778sup.zip › S0950268819001778sup001.docx]

Epidemiology and Infection

**Ceftiofur-resistant *Salmonella* *enterica* serovar Heidelberg of poultry origin - A risk profile using the Codex framework**

Carolee Carson, Xian-Zhi Li, Agnes Agunos, Daleen Loest, Brennan Chapman, Rita Finley, Manisha Mehrotra, Lauren M. Holden, Réjean Gaumond and Rebecca Irwin

**Supplementary Material:**

Supplementary Table S1: Relative ranking of the top five *Salmonella* serovars isolated from people around the world (1=most frequently isolated)

| **Rank** | **Canada**  **(2014-2016)** [1] | **United States**  **(2015)** [2] | **Africa**  **(2014)** [3] | **Americas**  **(2014)** [3] | **Asia**  **(2011)** [3] | **Europe**  **(2017)** [4] | **Oceania**  **(2013)** [3] | |
| --- | --- | --- | --- | --- | --- | --- | --- | --- |
| 1 | Enteritidis | Enteritidis | Enteritidis | Typhimurium | Enteritidis | Enteritidis | Typhimurium | |
| 2 | Typhimurium | Typhimurium | Typhimurium | Enteritidis | Infantis | Typhimurium | Enteritidis | |
| 3 | **Heidelberg** | Newport | Typhi | Panama | Saintpaul | Monophasic Typhimurium 1.4.[5].12:i:‐ | Infantis | |
| 4 | I 4,[5]12:i:- | Javiana | **Heidelberg** | I 4,[5],12:i:- | Typhimurium | Infantis | Brandenburg | |
| 5 | Newport | I 4,[5],12:i:- | Hadar | Saintpaul | Thompson | Newport | Typhi | |
| Data are the most recent available at the time of writing. | | | | | | | |  |

Supplementary Table S2: Annual relative frequency (%) of ceftiofur/ceftriaxone resistance among the most common *Salmonella* serovars isolated from humans and poultry in Canada (2003-2016)

|  | **Surveillance Year*** | | | | | | | | | | | | | |
| --- | --- | --- | --- | --- | --- | --- | --- | --- | --- | --- | --- | --- | --- | --- |
|  | **2003** | **2004** | **2005** | **2006** | **2007** | **2008** | **2009** | **2010** | **2011** | **2012** | **2013** | **2014** | **2015** | **2016** |
| **Percentage of isolates resistant to ceftiofur/ceftriaxone** | | | | | | | | | | | | | | |
| **Human Isolates** | | | | | | | | | | | | | | |
| All *Salmonella* | 6 | 7 | 6 | 4 | 2 | 2 | 3 | 5 | 7 | 7 | 8 | 5 | 5 | 4 |
| *S.* Heidelberg | 21 | 28 | 1 | 13 | 14 | 13 | 12 | 19 | 33 | 27 | 31 | 25 | 26 | 16 |
| *S.* Enteritidis | ≤ 1 | 0 | 0 | ≤ 1 | 0 | ≤ 1 | 0 | ≤ 1 | ≤ 1 | ≤ 1 | ≤ 1 | 0 | ≤ 1 | ≤ 1 |
| *S.* Typhimurium | 2 | ≤ 1 | ≤ 1 | 2 | ≤ 1 | 2 | 2 | 1 | 3 | 2 | 2 | ≤ 1 | 1 | 3 |
| **Retail Meat: Chicken** | | | | | | | | | | | | | | |
| All *Salmonella* | 32 | 43 | 11 | 10 | 10 | 13 | 22 | 22 | 30 | 26 | 25 | 21 | 13 | 7 |
| *S.* Heidelberg | 41 | 53 | 25 | 14 | 20 | 17 | 28 | 22 | 40 | 44 | 37 | 58 | 39 | 12 |
| *S.* Kentucky | 0 | 14 | 8 | 5 | 9 | 14 | 34 | 41 | 42 | 42 | 31 | 20 | 17 | 13 |
| *S.* Enteritidis | 0 | 0 | 0 | 0 | 0 | 0 | 0 | 0 | 0 | 0 | 0 | 0 | 0 | 0 |
| **Retail** **Meat: Turkey** | | | | | | | | | | | | | | |
| All *Salmonella* | **-** | **-** | **-** | **-** | **-** | **-** | **-** | **-** | **-** | 24 | 18 | 10 | 6 | 5 |
| *S.* Heidelberg | - | - | - | - | - | - | - | - | - | 39 | 29 | 29 | 17 | 22 |
| *S.* Enteritidis | **-** | **-** | **-** | **-** | **-** | **-** | **-** | **-** | **-** | 0 | 0 | 0 | 0 | 0 |
| *S.* Kentucky | **-** | **-** | **-** | **-** | **-** | **-** | **-** | **-** | **-** | **-** | 71 | 0 | 0 | 0 |
| *Canadian data from CIPARS 2003-2016 [5-18]; -, no surveillance data collected | | | | | | | | | | | | | | |

Supplementary Table S3: Ceftiofur/ceftriaxone-resistant *Salmonella* Heidelberg from retail poultry in Canada: three most common resistance patterns and percentage of isolates with resistance to three or more classes of antimicrobials

| **Sampled species/ product (sampling years)** | **Resistance patterns (%)** | **Percentage of isolates with resistance to three or more classes of antimicrobials** |
| --- | --- | --- |
| Chicken  (2003-2016) | A2C-AMP-CRO (90%)  A2C-AMP-CRO-STR (4%)  AMC-AMP-TIO-CRO (2%) | 3% |
| Turkey  (2011-2016) | A2C-AMP-CRO (69%)  A2C-AMP-CRO-TET (9%)  A2C-AMP-CRO-STR-SSS-TET (7%) | 9% |
| Frozen raw breaded chicken products  (2011-2014) | A2C-AMP-CRO (87%)  AMC-AMP-TIO-CRO (6%)  A2C-AMP-CRO-STR (4%) | 2% |
| Abbreviations: A2C=amoxicillin-clavulanic acid, cefoxitin and ceftiofur; AMC=amoxicillin-clavulanic acid; AMP=ampicillin; CRO=ceftriaxone; SSS=sulfisoxazole; STR=streptomycin; TET=tetracycline; TIO=ceftiofur  Data source: Canadian Integrated Program for Antimicrobial Resistance Surveillance. Personal communication. | | |

Supplementary Table S4: Burden of illness summary for antimicrobial-resistant *Salmonella*

| **Serovar**  **Year (Location)** | **Study type** | **Burden of illness measures** | **Findings** | **Reference** |
| --- | --- | --- | --- | --- |
| *Salmonella* Newport  1999-2001 (USA) | Retrospective case-control study | Symptoms and hospitalization | Patients infected with multi-drug resistant (including resistance to ceftiofur and ceftriaxone) *S.* Newport were more likely to have bloody diarrhea (OR 4.7; CI 1.4-17.9) than those infected with pan-susceptible S. Newport. No differences in hospitalization rates, or the incidence of fever, vomiting or cramps were demonstrated. | [19] |
| Non-typhoidal *Salmonella* (NTS)  2003-2013 (USA) | Case Control | Incidence of bacteremia | Infections caused by resistant NTS were associated with higher incidences of bacteremia (OR 1.84; CI 1.57-2.15), especially in individuals older than 64 years | [20] |
| NTS  2004-2009 (USA) | Cohort | Hospitalization | Higher hospitalization rates when infected with resistant NTS (OR 1.98; CI 1.3-3.02) | [21] |
| NTS  1996-2007 (USA) | Cohort | Bloodstream infection | Higher incidence of bacteremia with infections caused by resistant NTS (OR 1.38; CI 1.1-1.7) | [22] |
| *Salmonella*  1971-1983 (USA) | Literature review | Mortality rates | Of 52 outbreaks, patients infected with resistant *Salmonella* had a 4% case fatality rate, vs. 0.2% for those infected with susceptible *Salmonella* | [23] |
| NTS  1984-2002 (USA) | Outbreak analysis | Hospitalization and mortality | Hospitalization rates were higher with outbreaks caused by resistant NTS outbreaks (22%) than with susceptible NTS outbreaks (8%) (*p*<0.01). Mortality rates in resistant NTS outbreaks were 0.1%, vs. 0.06% in susceptible NTS outbreaks (not a significant difference *p*=0.57) | [24] |
| *S.* Heidelberg  1987 (United Kingdom) | Cost analysis | Lost productive output (time off work) and hospital costs | A hospital outbreak of multidrug-resistant *S.* Heidelberg affected 17 patients and 2 staff costing the hospital ca. £21000. Staff disruption included 167 person hours. | [25] |
| *S.* Typhimurium  1995-1999  (Denmark) | Matched cohort study | Mortality rates | Compared to the general Danish population, patients with susceptible *S.* Typhimurium infections were 2.3 times more likely to die 2 years after infection. Patients infected with strains resistant to ampicillin, chloramphenicol, streptomycin, sulfonamide, and tetracycline were 4.8 times more likely to die, and those with quinolone resistant strains were 10.3 times more likely to die. | [26] |
| *S.* Typhimurium  1995-2000  (Denmark) | Registry-based cohort study | Mortality rates, risk of invasive illness | The risk of invasive infection or death within 90 days of infection with quinolone-resistant *S.* Typhimurium was 3.15-fold higher, compared to infections by pan-susceptible strains (*p*=0.0058). | [27] |
| NTS  1996-2001 (USA) | Surveillance analysis | Bloodstream infection, hospitalization and length of hospital stay | Infections with resistant NTS were associated with increased incidence of bacteremia and hospitalization. Median hospital stay was 4 and 3 days, respectively, for patients with resistant and susceptible infection. | [28] |
| S. Choleraesuis  1996-2004 (Taiwan) | Retrospective cohort study | Mortality, recurrence, invasive infection | Bacteremia due to ciprofloxacin-resistant isolates had a 13% fatality rate, compared to 6% of those infected with ciprofloxacin-susceptible isolates. However, 30% ciprofloxacin-resistant isolates were associated with invasive disease, vs. 7% of the susceptible isolates. The susceptible isolates also tended to cause more recurrent infections. | [29] |
| *S.* Typhimurium  1999-2000  (Canada) | Case-comparison analysis | Hospitalization | Hospitalization was more likely (OR 2.3) with an isolate resistant to at least ampicillin, chloramphenicol and/or kanamycin, streptomycin, sulphamethoxazole, and tetracycline, compared to pan-susceptible isolate. | [30] |
| *S.* Newport  2002-2003 (USA) | Case-comparison analysis | Hospitalization rates and duration, nature and duration of symptoms | No significant differences in symptoms, duration of symptoms, hospitalization, or duration of hospitalization were demonstrated between those infected with multi-drug resistant vs. pan-susceptible S. Newport. | [31] |
| NTS  2003-2007 (USA) | Surveillance analysis | Hospitalization and mortality | Resistant *S.* Typhimurium cases were hospitalized more frequently (42%) than susceptible cases (25%). The case fatality rate for resistant infections was 3.8%, and 0.3% for susceptible cases, however, after age adjustment the significance of the difference decreased (*p*=0.07) | [32] |
| NTS  2003-2008  (Hong Kong) | Retrospective cohort study | Duration of hospitalization, hospital costs, and mortality rate | Duration of hospitalization was 1 day longer for patients infected with quinolone resistant NTS, compared to those infected with quinolone susceptible NTS.  Resistant infections had up to 43% more costly hospital stays than susceptible cases. Differences in mortality were not statistically significant. | [33] |
| NTS  2006-2008 (USA) | Multi-center cohort study | Bloodstream infection, hospitalization, duration of hospitalization | Depending on the resistance pattern of the NTS isolates, the risk of hospitalization >3 days were up to 4.1 times higher, and the risk for bloodstream infection was 2.2-10 times higher among patients infected with resistant NTS, compared to those infected with pan-susceptible NTS. | [34] |

Supplementary Table S5: Summary of data quality, level of concern, data gaps, and potential value of whole genome sequencing

| **Sections** | | **Data available (Y/N/S/L^#^)** | **New Data Needed to Make Risk Management Decision?** |
| --- | --- | --- | --- |
| 1. Description of the AMR food safety issue (per Codex definition) | | Data Quality Score*: 12; Level of Concern: 3 | |
| 2. Information on the AMR organism /determinant | | Data Quality Score: 12; Level of Concern: 3 | |
| 2.1. Characteristics of *S.* Heidelberg | 2.1.1. Sources and transmission routes | Y | No. Other foodborne sources of *S.* Heidelberg may not be sampled by CIPARS, however we believe the predominant sources are captured. |
|  | 2.1.2. Pathogenicity of *S.* Heidelberg, Virulence, and Linkages to Resistance | S | More data are needed on the genetic linkages of pathogenicity, resistance, and virulence characteristics, and it impacts human illness. Information from WGS would be beneficial. |
|  | 2.1.3. Distribution, frequency and concentrations of CSH in the food chain | Y | CIPARS data provided in this risk profile are based on the sampling of chicken (skin-on legs and wings from routine surveillance and a CIPARS targeted study of chicken nuggets/strips) and ground turkey, however the hazard could be present in fresh breasts, legs and wings (skinless), thighs or other more processed chicken products such as chicken burgers. Surveillance of such additional chicken retail products at regional sentinel sites is conducted by FoodNet Canada, and combining this data with CIPARS data will be of value to enhance knowledge of particular chicken retail products. Sampling of imported chicks (or post hatching of imported eggs) could enhance knowledge of non-domestic sources. |
|  | 2.1.4. Growth and survivability, including inactivation in foods (D-value, minimum pH for growth) of CSH in the poultry production- to-consumption continuum | L | No, not specifically needed for CSH, though the information would be useful for *Salmonella* control in general and useful for quantitative modelling. |
| 2.2. Characteristics of ceftiofur resistance in *Salmonella* sp. | 2.2.1. Cephalosporin resistance mechanisms and location of the resistance determinants | Y | No. The fact that resistance is often found on a mobile plasmid, which can be exchanged between bacterial species, is sufficient to conclude that this is transmissible resistance |
|  | 2.2.2. Cross-resistance and/or co-resistance to other antimicrobial agents | Y | Even though phenotypic data exist in Canada, knowledge of the genes involved in cross-resistance/co-selection would assist decision making about interventions (focused on ceftiofur or include other antimicrobials). Information from WGS would be beneficial |
|  | 2.2.3. Transferability of resistance determinants between microorganisms | Y | Transfer has been proven, however more information could be gathered about the rate of transfer. |
| 3. Information on the antimicrobial agent(s) to which resistance is expressed - 3rd generation cephalosporins | | Data Quality Score: 12; Level of Concern: 3 | |
| 3.1. Class of the antimicrobial agent(s) |  | Y | No |
| 3.2. Non-human uses of 3rd generation cephalosporins | 3.2.1. Formulation of 3rd generation cephalosporins | Y | No |
|  | 3.2.2. Distribution, cost (in Canadian dollars) and availability of ceftiofur | S | The quantities of 3^rd^ generation cephalosporins being imported for ‘own use’ or as active pharmaceutical ingredients are unknown. New regulations by Health Canada will improve this data source as of 2019 [35] |
|  | 3.2.3. Purpose and use of ceftiofur (in feed, food animals, crop production and/or during food processing) AND Potential extra-label/off label, use of approved antimicrobial agent(s) and use of non-approved ceftiofur | Y | Having inclusion of more broiler chicken and turkey farms (with data that reach back to the hatcheries) in the CIPARS program would enhance our knowledge of ceftiofur use in hatcheries and breeders. |
|  | 3.2.4. Methods, routes of administration of ceftiofur and frequency | Y |  |
|  | 3.2.5. Potential role of cross-resistance or co-resistance with use of other antimicrobial agent(s) in food production | Y |  |
|  | 3.2.6. Trends in the use of ceftiofur in poultry and information on emerging resistance in poultry and poultry meat | Y |  |
|  | 3.2.7. Alternative antimicrobials in the livestock commodity | Y | No |
|  | 3.2.8. Information on the relationship between the use of the antimicrobial agent(s) and the occurrence of resistant microorganisms or resistance determinants in the food commodity of concern | S | CIPARS has Canadian data on use and resistance, and can demonstrate similar temporal trends between use and AMR. CIPARS needs to conduct the epidemiological modelling to determine and measure the association between the two. |
| 3.3 Human uses | 3.3.1 Spectrum of activity and indications for treatment | Y | No |
|  | 3.3.2 Importance of the antimicrobial agent, including consideration of critically important antimicrobial lists | Y | No |
|  | 3.3.3 Distribution, cost and availability | Y | No |
|  | 3.3.4 Availability of alternative antimicrobial agent(s) | Y | No |
|  | 3.3.5 Trends in the use of the antimicrobial agent(s) in humans and information on emerging diseases due to microorganism(s) resistant to the antimicrobial agent(s) or classes. | L | Specific quantitative data regarding disease manifestations related to CSH in humans are needed |
| 4. Information on the Food Commodity – chicken and turkey | | Data Quality Score: 8; Level of Concern: 3 | |
| 4.1 Source(s) (domestic or imported), production volume, distribution and per capita consumption of foods or raw materials identified with the AMR hazard(s) of concern. |  | Y | There is a large data gap on the concentration of CHS (# colony forming units/gram of material) on all poultry retail products and at all stages in the production-to-consumption continuum. |
|  | 4.1.1 Characteristics of the food product(s) that may impact risk management (e.g., further processed, consumed cooked, pH, water activity, etc.) | Y |  |
|  | 4.1.2 Description of the food production to consumption continuum (e.g., primary production, processing, storage, handling, distribution and consumption) and the risk factors that affect the microbiological safety of the food product of concern. | Y | No |
| 5. Information on adverse public health effects | | Data Quality Score: 8.5; Level of Concern: 3 | |
| 5.1 Characteristics of the disease caused by the identified foodborne AMR microorganisms or by pathogens that have acquired resistance determinants via food | 5.1.1. Trends in AMR foodborne disease | Y | Data gap exists regarding the burden of illness and number of cases attributable to CSH in Canada, related to the difference between reported numbers and true incidence (although an estimation approach have been published to account for unreported cases). |
|  | 5.1.2. Frequency and severity of effects including case-fatality rate, hospitalisation rate and long-term complications | S | Need current Canada-specific data with regards to the impact of ceftiofur/ceftriaxone resistance of *S.* Heidelberg on the frequency and severity of infections. |
|  | 5.1.3. Susceptible population and risk factors | Y | No |
|  | 5.1.4. Epidemiological pattern (outbreak or sporadic) | Y | No |
|  | 5.1.5. Regional, seasonal and ethnic differences in the incidence of foodborne disease due to AMR hazard(s) | Y | No |
|  | 5.1.6. Additional information on the relationship between the presence of the AMR microorganisms or determinants in the food commodity and the occurrence of the adverse health effect(s) in humans | N | While information exists for one province and one year, determining genetic linkages between CSH in poultry and CSH in human clinical cases will be valuable for definitive source attribution. Information from WGS would be beneficial. |
| 5.2 Consequences of resistance on the outcome of the disease: Increased frequency and severity of infections, including prolonged duration of disease, loss of treatment options and treatment failures, increased frequency of bloodstream infections, hospitalization and mortality |  | Y | Need current Canadian-specific data regarding treatment failures and limited treatment options specifically due to resistance |
| 6. Risk management options | | Data Quality Score: 12; Level of Concern: N/A | |
| 6.1. Identification of risk management options to control the AMR hazard along the production to consumption continuum both in the pre-harvest and post-harvest stages | 6.1.1. Measures to reduce the risk related to the selection and dissemination of foodborne AMR microorganisms(s) | Y | For the selection of *S*. Heidelberg isolates resistant to ceftiofur, CIPARS should conduct quantitative epidemiological modeling of AMU and AMR with the sentinel farm data. |
|  | 6.1.2. Measures to minimize the contamination and cross-contamination of food by AMR microorganism(s) | L | A qualitative description of what might be risk management options is available; quantitative data supporting the effectiveness of these measures is lacking. |
| 6.2 Effectiveness of current management practices in place based on surveillance data or other sources of information |  | L | Evaluation of the effectiveness of risk management interventions is needed. WGS can be valuable in determining the impact of farming practices on AMR, virulence, and survival. |
| N/A – not applicable; *Data quality score: data were only scored as it pertains to risk, background information were not scored; #: Y-Yes, N-No, S-Some, L-Limited | | | |

**References**

1. **Government of Canada.** National Enteric Surveillance Program (NESP) Annual Summary 2016. 2018; Available at: <http://publications.gc.ca/collections/collection_2018/aspc-phac/HP37-15-2016-eng.pdf>. Accessed 07/01, 2019.

2. **Centers for Disease Control and Prevention.** National Enteric Disease Surveillance: *Salmonella* Annual Report 2015. 2017; Available at: <https://www.cdc.gov/nationalsurveillance/pdfs/2015_SalmonellaREPORT-508.pdf>. Accessed 07/18, 2019.

3. **World Health Organization.** Global Foodborne Infections Network 2009 and 2011. 2014; Available at: <http://www.who.int/gfn/activities/en/#databank.> Accessed August 31, 2014.

4. **European Food Safety Authority and European Centre for Disease Prevention and Control.** The European Union summary report on trends and sources of zoonoses, zoonotic agents and food-borne outbreaks in 2017. *EFSA Journal*16, 5500. EFSA Journal 2018;16:5500.

5. **Government of Canada.** Canadian Integrated Program for Antimicrobial Resistance Surveillance (CIPARS) 2003 Annual Report. Available at: <http://publications.gc.ca/collections/Collection/H39-1-3-2003E.pdf>. Accessed 07/18, 2019.

6. **Government of Canada.** Canadian Integrated Program for Antimicrobial Resistance Surveillance (CIPARS) 2004 Annual Report. Available at: <http://publications.gc.ca/collections/Collection/HP2-4-2004E.pdf>. Accessed 07/18, 2019.

7. **Government of Canada.** Canadian Integrated Program for Antimicrobial Resistance Surveillance (CIPARS) 2005 Annual Report. Available at: <http://publications.gc.ca/collections/collection_2008/phac-aspc/HP2-4-2005E.pdf>. Accessed 07/18, 2019.

8. **Government of Canada.** Canadian Integrated Program for Antimicrobial Resistance Surveillance (CIPARS) 2006 Annual Report. Available at: <http://publications.gc.ca/collections/collection_2009/aspc-phac/HP2-4-2006E.pdf>. Accessed 07/18, 2019.

9. **Government of Canada.** Canadian Integrated Program for Antimicrobial Resistance Surveillance (CIPARS) 2007 Annual Report. Available at: <http://publications.gc.ca/collections/collection_2010/aspc-phac/HP2-4-2007-eng.pdf>. Accessed 07/18, 2019.

10. **Government of Canada.** Canadian Integrated Program for Antimicrobial Resistance Surveillance (CIPARS) 2008 Annual Report. Available at: <https://www.canada.ca/en/public-health/services/surveillance/canadian-integrated-program-antimicrobial-resistance-surveillance-cipars/cipars-2008-annual-report.html>. Accessed 07/18, 2019.

11. **Government of Canada.** Canadian Integrated Program for Antimicrobial Resistance Surveillance (CIPARS) 2009 Annual Report. Available at: <http://publications.gc.ca/collections/collection_2013/aspc-phac/HP2-4-2009-eng.pdf>. Accessed 07/18, 2019.

12. **Government of Canada.** Canadian Integrated Program for Antimicrobial Resistance Surveillance (CIPARS) 2010 Annual Report. Available at: <http://publications.gc.ca/collections/collection_2014/aspc-phac/HP2-4-2010-eng.pdf>. Accessed 07/18, 2019.

13. **Government of Canada.** Canadian Integrated Program for Antimicrobial Resistance Surveillance (CIPARS) 2011 Antimicrobial Resistance Short Report. Available at: <http://publications.gc.ca/collections/collection_2013/aspc-phac/HP2-4-2-2011-eng.pdf>. Accessed 07/18, 2019.

14. **Government of Canada.** Canadian Integrated Program for Antimicrobial Resistance Surveillance (CIPARS) 2012 Annual Report. Available at: <https://www.canada.ca/en/public-health/services/surveillance/canadian-integrated-program-antimicrobial-resistance-surveillance-cipars/canadian-integrated-program-antimicrobial-resistance-surveillance-cipars-annual-report/cipars-2012-annual-report.html>. Accessed 07/18, 2019.

15. **Government of Canada.** Canadian Integrated Program for Antimicrobial Resistance Surveillance (CIPARS) 2013 Annual Report. Available at: <https://www.canada.ca/en/public-health/services/surveillance/canadian-integrated-program-antimicrobial-resistance-surveillance-cipars/cipars-2013-annual-report.html>. Accessed 07/18, 2019.

16. **Government of Canada.** Canadian Integrated Program for Antimicrobial Resistance Surveillance (CIPARS) 2015 Annual Report. Available at: <https://www.canada.ca/en/public-health/services/surveillance/canadian-integrated-program-antimicrobial-resistance-surveillance-cipars/2015-annual-report-summary.html>. Accessed 07/18, 2019.

17. **Government of Canada.** Canadian Integrated Program for Antimicrobial Resistance Surveillance (CIPARS) 2016 Annual Report. Available at: <http://publications.gc.ca/collections/collection_2018/aspc-phac/HP2-4-2016-eng.pdf>. Accessed 07/18, 2019.

18. **Government of Canada.** Canadian Integrated Program for Antimicrobial Resistance Surveillance (CIPARS) 2014 Annual Report. Available at: <https://www.canada.ca/en/public-health/services/surveillance/canadian-integrated-program-antimicrobial-resistance-surveillance-cipars/cipars-2014-annual-report-summary.html>. Accessed 07/18, 2019.

19. **Gupta A, Fontana J, Crowe C, Bolstorff B, Stout A, Duyne SV, et al.** Emergence of multidrug-resistant *Salmonella enterica* serotype Newport infections resistant to expanded-spectrum cephalosporins in the United States. J Infect Dis 2003;188(11):1707-1716.

20. **Angelo KM, Reynolds J, Karp BE, Hoekstra RM, Scheel CM, Friedman C.** Antimicrobial resistance among nontyphoidal *Salmonella* isolated from blood in the United States, 2003–2013. J Infect Dis 2016;214(10):1565-1570.

21. **Russell Barlow.** Emerging nontyphoidal *Salmonella* antibiotic resistance in Oregon 2004-2009: risk factors and trends. Oregon: Oregon Health and Science University: OHSU Digital Commons; 2012.

22. **Crump JA, Medalla FM, Joyce KW, Krueger AL, Hoekstra RM, Whichard JM, et al.** Antimicrobial resistance among invasive nontyphoidal *Salmonella enterica* isolates in the United States: National Antimicrobial Resistance Monitoring System, 1996 to 2007. Antimicrob Agents Chemother 2011 Mar;55(3):1148-1154.

23. **Holmberg SD, Wells JG, Cohen ML.** Animal-to-man transmission of antimicrobial-resistant *Salmonella*: investigations of U.S. outbreaks, 1971-1983. Science 1984;225(4664):833-835.

24. **Varma JK, Greene KD, Ovitt J, Barrett TJ, Medalla F, Angulo FJ.** Hospitalization and antimicrobial resistance in *Salmonella* outbreaks, 1984-2002. Emerg Infect Dis 2005;11(6):943-946.

25. **Barnass S, O'Mahony M, Sockett PN, Garner J, Franklin J, Tabaqchali S.** The tangible cost implications of a hospital outbreak of multiply-resistant *Salmonella*. Epidemiol Infect 1989;103(2):227-234.

26. **Helms M, Vastrup P, Gerner-Smidt P, Molbak K.** Excess mortality associated with antimicrobial drug-resistant *Salmonella* Typhimurium. Emerg Infect Dis 2002;8(5):490-495.

27. **Helms M, Simonsen J, Molbak K.** Quinolone resistance is associated with increased risk of invasive illness or death during infection with *Salmonella* serotype Typhimurium. J Infect Dis 2004;190(9):1652-1654.

28. **Varma JK, Molbak K, Barrett TJ, Beebe JL, Jones TF, Rabatsky-Ehr T, et al.** Antimicrobial-resistant nontyphoidal *Salmonella* is associated with excess bloodstream infections and hospitalizations. J Infect Dis 2005;191(4):554-561.

29. **Wang JY, Hwang JJ, Hsu CN, Lin LC, Hsueh PR.** Bacteraemia due to ciprofloxacin-resistant *Salmonella enterica* serotype Choleraesuis in adult patients at a university hospital in Taiwan, 1996-2004. Epidemiol Infect 2006;134(5):977-984.

30. **Martin LJ, Fyfe M, Dore K, Buxton JA, Pollari F, Henry B, et al.** Increased burden of illness associated with antimicrobial-resistant *Salmonella enterica* serotype Typhimurium infections. J Infect Dis 2004;189(3):377-384.

31. **Devasia RA, Varma JK, Whichard J, Gettner S, Cronquist AB, Hurd S, et al.** Antimicrobial use and outcomes in patients with multidrug-resistant and pansusceptible *Salmonella* Newport infections, 2002-2003. Microb Drug Resist 2005;11(4):371-377.

32. **Solghan SM, Dumas NB, Root TP, Quinlan TM, Armstrong LR, Spina NL, et al.** Multidrug-resistant nontyphoidal *Salmonella* in New York state's foodborne diseases active surveillance network counties. Foodborne Pathog Dis 2010;7(2):167-173.

33. **Broughton EI, Ip M, Coles CL, Walker DG.** Higher hospital costs and lengths of stay associated with quinolone-resistant *Salmonella* *enterica* infections in Hong Kong. J Public Health 2010;32(2):165-172.

34. **Krueger AL, Greene SA, Barzilay EJ, Henao O, Vugia D, Hanna S, et al.** Clinical outcomes of nalidixic acid, ceftriaxone, and multidrug-resistant nontyphoidal S*almonella* infections compared with pansusceptible infections in FoodNet sites, 2006–2008. Foodborne Pathog Dis 2014;11(5):335-341.

35. **Government of Canada.** Regulations Amending the Food and Drug Regulations (Veterinary Drugs-Antimicrobial Resistance). 2017(SOR/2017-76 May 5, 2017).
