# Supplementary figures and images for: Ceftiofur-resistant Salmonella enterica serovar Heidelberg of poultry origin – a risk profile using the Codex framework
Source: Epidemiol Infect. 2019 Nov 4;147:e296. doi: 10.1017/S0950268819001778 (PMC6836576; doi:10.1017/S0950268819001778)

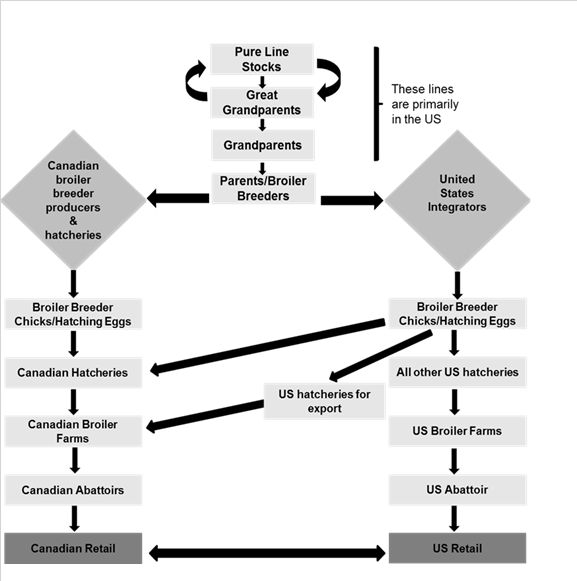

Supplement: Supplementary file 1 [file S0950268819001778sup.zip › S0950268819001778sup002.tif]
